# Challenges With the Use of Digital Sham: Systematic Review and Recommendations

**DOI:** 10.2196/44764

**Published:** 2023-10-24

**Authors:** Ernst Bos, Katrin H Preller, Gavneet Kaur, Pooja Malhotra, Saifuddin Kharawala, Dario Motti

**Affiliations:** 1 F Hoffmann-La Roche Ltd Basel Switzerland; 2 Bridge Medical Consulting Limited London United Kingdom

**Keywords:** cognitive behavioral therapy, digital intervention, sham, neurosciences, neurodegenerative diseases, placebo, psychiatric disorders, systematic review, mobile phone

## Abstract

**Background:**

Digital therapeutics (DTx) are software-based products that prevent, manage, or treat a medical condition and are delivered through a smartphone app, web application, or wearable device. Clinical trials assessing DTx pose challenges, foremost among which is designing appropriate *digital shams* (or *digital placebos*), which should ideally mimic DTx (in terms of design, components, and duration of treatment) while omitting the active principle or component.

**Objective:**

The objective of our review was to understand how digital shams are being used in clinical research on DTx in neuroscience, which is the most common therapy area for DTx.

**Methods:**

We conducted a systematic literature review of DTx in neuroscience (including neurodevelopmental, neurodegenerative, and psychiatric disorders) with a focus on controlled clinical trials involving digital shams. Studies were identified from trial registries (ClinicalTrials.gov, the European Union Clinical Trials Register, and Trial Trove) and through structured searches in MEDLINE and Embase (both via the Embase website) and were limited to articles in English published from 2010 onward. These were supplemented by keyword-based searches in PubMed, Google, and Google Scholar and bibliographic searches. Studies assessing DTx in neuroscience (including neurodevelopmental, neurodegenerative, and psychiatric disorders) were included. Details related to the publication, DTx, comparator, patient population, and outcomes were extracted and analyzed.

**Results:**

Our search criteria identified 461 neuroscience studies involving 213 unique DTx. Most DTx were extended reality based (86/213, 40.4%) or mobile device based (56/213, 26.3%); 313 were comparative, of which 68 (21.7%) used shams. The most common therapeutic areas assessed in these studies were stroke (42/213, 19.7%), depression (32/213, 15%), and anxiety (24/213, 11.3%). The most common treatments were cognitive behavioral therapy or behavioral therapy (67/213, 32.4%), physical rehabilitation (60/213, 28.2%), and cognitive training (41/213, 19.2%). We identified the following important issues related to the use of digital shams in neuroscience: shams were not validated before use in studies, they varied widely in design (from being nearly identical to the DTx to using different software programs altogether), and the level of patient engagement or satisfaction with the sham and the impact of the sham on study outcomes were infrequently reported.

**Conclusions:**

Digital shams are critical for the clinical development of DTx in neuroscience. Given the importance of sham controls in evaluating DTx efficacy, we provide recommendations on the key information that should be reported in a well-designed DTx trial and propose an algorithm to allow the correct interpretation of DTx study results. Sham-controlled studies should be routinely used in DTx trials—in early-phase studies—to help identify DTx active components and—in late-phase studies—to confirm the efficacy of DTx. The use of shams early in development will ensure that the appropriate sham control is used in later confirmatory trials.

## Introduction

Digital therapeutics (DTx), a new frontier in medicine, are software-based products that prevent, manage, or treat a medical condition [1-4]. DTx are generally delivered through a smartphone app, web application, or wearable device and may use extended reality (XR) or resemble a computer game [1-6]. The explosion of interest in DTx has coincided with the popularity of social networks, the ubiquity of smartphones and wearables, and the increasing sophistication of cloud-based data platforms [1]. The COVID-19 pandemic may have further driven interest in DTx. DTx may not only improve access to therapies with established clinical benefits (such as cognitive behavioral therapy [CBT]) but may also open new areas of intervention altogether. In neuroscience, DTx are being explored in motor rehabilitation and cognitive rehabilitation in patients with stroke, Parkinson disease, and multiple sclerosis (MS) as well as in depression, fatigue, and chronic pain [4,7]. Outside neuroscience, DTx are being developed for conditions such as gastrointestinal disorders, diabetes, heart failure, asthma, and chronic obstructive pulmonary disease [8].

For DTx that do not fall under the United States Food and Drug Administration enforcement discretion, standard regulatory requirements for class I, II, and III devices apply [8,9].

To appropriately study the effectiveness of DTx, it is crucial to establish clear control groups, including sham (or placebo) controls. There is no specific United States Food and Drug Administration guidance on the use of digital shams; however, several recently approved DTx have successfully used them in studies [1,3]. Similarly, there is no specific regulatory guidance in Europe, although issues with designing a digital sham have been identified as a regulatory challenge [8,9].

The formulation of these control arms should consider several factors that contribute to therapeutic efficacy in DTx trials. These factors include the following:

- *Nonspecific factors*: These encompass elements such as the expectation of improvement and regression to the mean, which can influence trial outcomes.
- *Engagement-related factors*: Factors arising from engagement with the DTx, such as self-monitoring, can also impact trial results.
- *Active component of the DTx*: This is the specific therapeutic element of the DTx, such as CBT delivered through a digital platform.

In traditional clinical trials, a placebo control consists of an inert substance that may improve outcomes because of nonspecific effects such as regression to the mean, but it lacks an active ingredient, whereas an active control includes both the nonspecific factors and active ingredient.

DTx trials differ from traditional clinical trials owing to the presence of therapeutic factors arising from engagement with digital intervention. Therefore, in DTx trials, a placebo control or digital sham would need to include not only the same nonspecific factors but also the same engagement-related factors as the DTx itself, with only the active component excluded. Similarly, an active control for DTx would be a treatment that incorporates comparable nonspecific and engagement-related factors and includes an active component that differs from the active component of the DTx being studied. Properly formulating and implementing these control groups is essential for the reliable assessment of DTx effectiveness and for distinguishing the specific therapeutic effects of the digital intervention from nonspecific and engagement-related factors.

Given this scenario, designing and interpreting the results of DTx clinical trials poses several challenges. The first step involves identifying the fundamental active component of a DTx separately from the engagement-related factors in the intervention, which can be difficult. Second, designing a digital sham by removing the active component alone can be difficult. In the case of a DTx that involves CBT delivered via a video game, for example, a simple non–CBT-based psychoeducational sham control may serve the purpose of removing the active component; however, if it is significantly less engaging than the DTx and the variation in engagement levels could potentially introduce confounding into the study results. Such a study may demonstrate efficacy for the DTx even if the CBT component is ineffective (ie, it yields a false-positive result). Conversely, if the sham includes aspects of the active component, then such a sham-controlled study may fail despite the DTx being effective (ie, yielding a false-negative result) [10-12]. The corollary of this is that interpreting a sham-controlled study is not straightforward. If a study fails to demonstrate the efficacy of the DTx, this may be because of a lack of efficacy of the DTx or because of an inappropriate sham control that included active components and thus masked the DTx effect. Conversely, a sham-controlled study may succeed in demonstrating DTx efficacy either because of the real efficacy of the DTx or because of an inappropriate sham control that was substantially less engaging than the DTx.

An ideal DTx confirmatory trial would involve an appropriately designed sham control that has been validated, that is, has been demonstrated to, or can reasonably be expected to, have a comparable impact on nonspecific and engagement-related factors, such as the DTx itself.

Given the complexities involved in DTx clinical trials, especially with reference to the use of appropriate sham controls, it is important to better understand how digital sham controls have been implemented in these trials and how their design and validation have been reported.

Thus, DTx are likely to shape the future of medicine. Therefore, it is important to understand how digital shams are being used in clinical research on DTx in neuroscience, which is the most common therapy area for DTx [4,7,8]. Recently, Lutz et al [13] (nonsystematically) reviewed the control conditions in DTx trials. Although this review was limited to DTx listed in the Digital Therapeutics Alliance product list with findings based on only 14 randomized controlled trials, it also highlighted important challenges in defining sham controls for DTx [13]. There remains a need for a more comprehensive review that explores all available clinical trials on DTx in neuroscience. Here, we describe a systematic literature review of DTx in neuroscience (including neurodevelopmental, neurodegenerative, and psychiatric disorders) with a focus on controlled clinical trials, particularly those involving digital shams. To the best of our knowledge, the use of digital shams in DTx clinical trials has not yet been systematically examined. We hope that the results will help establish a scientific road map for evidence-based DTx development and drive the formulation of best practices and guidelines for research, ensuring the correct evaluation of patient benefit and safety.

The research questions addressed in the literature review were as follows. In the field of neuroscience (including neurodevelopmental, neurodegenerative, and psychiatric disorders):

1. How has digital sham been used in clinical research?
2. What proportion of DTx studies involved digital sham?
3. What was the objective of using digital sham in the study or in the larger developmental plan of the manufacturer?
4. What was the nature of digital sham used in these studies? Was the digital sham validated before its use in clinical trials?
5. How did digital sham compare with the active DTx interventions in these studies? Which aspects were common in both treatment arms, and which aspects differed, in terms of nature of intervention, number of hours of treatment, level or intensity of patient engagement, etc?
6. What were the compliance or dropout rates with the use of digital sham? How did those compare with the compliance or dropout rates with active intervention?
7. What were the results of these studies? What were the effect sizes (change from baseline) with the use of digital sham, across various outcomes? What were the differences in effect sizes between active DTx intervention and digital sham?
8. What challenges were identified in the design, development, and use of digital sham? What modifications or improvements were proposed?

## Methods

### Search Strategy

Studies were identified from trial registries (ClinicalTrials.gov [14], the European Union Clinical Trials Register [15], and Trial Trove [16]) and through structured searches in MEDLINE and Embase (both via the Embase website). The search strategy used a facet for DTx and digital shams and was limited to articles in English published from 2010 onward (refer to the Embase criteria in Table S1 in Multimedia Appendix 1 [17-45] and the final search strategy in Table S2 in Multimedia Appendix 1). Structured searches (run on May 10, 2021) were supplemented by additional searches conducted in November 2021. The additional supplementary searches included keyword-based searches in PubMed, Google, and Google Scholar to identify comparative studies involving DTx and digital shams in neuroscience and a review of bibliographies of relevant publications identified by the structured searches to identify further relevant citations.

Abstracts and titles were screened by a single reviewer who then examined the full text in detail. A quality check was conducted by a second reviewer who confirmed the accuracy of all the steps included in screening; this covered approximately 20% of all screening decisions.

### Selection Criteria

Studies that assessed DTx in neuroscience (including neurodevelopmental, neurodegenerative, and psychiatric disorders) were included. Studies that assessed DTx in nonneuroscience indications (such as respiratory disorders, metabolic disorders, and cardiovascular disorders) were excluded. Study designs of interest were clinical trials. All observational studies were excluded. There were no restrictions on age, ethnicity, outcome, and geography. These selection criteria were applied to the structured and supplementary searches.

### Definitions

DTx, as per the Digital Therapeutics Alliance definition, is a product that is driven by high-quality software programs and that prevents, manages, or treats a medical disorder or disease.

In the absence of a universally accepted definition of digital sham, we operationally defined it as a comparator designed to mimic the DTx (eg, with a similar design, components, and duration of treatment as the DTx) but with the DTx active principle or component being removed or reduced in intensity [46].

DTx were categorized according to the treatment delivery techniques used. These include the following:

- *Mobile devices*: These include DTx that are delivered through software applications. Examples include Daylight, reSET-O, Quit genius, and EndeavorRx.
- *Computer based*: These include DTx that are training modules, psychoeducation, etc delivered through a computer. Examples include the Brain HQ, Cogmed, Cogniplus, and CogPack. These also include video games delivered through a computer. Examples include SPARX (Smart, Positive, Active, Realistic, X-factor) and Dr Kawashima’s Brain Training.
- *XR based*: These include DTx that use XR as the mode of delivery. Examples include Kinect-based virtual reality games, EaseVRx, and Reh@City.
- *Wearables*: These include wearable devices (smartwatch, wristband, headband, etc) that measure physiological data and provide stimulation, vibration, or a signal to treat the problem. For example, The NightWare program is an app that works on a smartwatch and is therefore classified as wearable. Other examples include Relivion and Rapael Smart Glove.
- *Web based*: These include web-based and internet-based DTx. Examples include SHUTi, Deprexis, and FitMindKit.

### Data Analysis

Details related to the publication, DTx, comparator, patient population, and outcomes were extracted and analyzed. Studies were categorized based on the efficacy results of DTx and sham, and this categorization was used to inform a future analytical framework. Groups 1 and 2 included studies in which there was no significant improvement from baseline in the primary outcome in the sham control arm. In group 1, both DTx and sham arms failed to show improvement from baseline (DTx− and Sh−), whereas group-2 studies successfully demonstrated the efficacy of DTx (DTx+ and Sh−). In group-2 studies, it is pertinent to examine whether the comparative efficacy of the DTx arm may have occurred because of inappropriate sham control. Groups 3 and 4 included studies in which there was significant improvement from baseline with sham. Group 3 included studies that failed despite the DTx showing improvement from baseline (DTx+, Sh+ [note that DTx was not significantly inferior to sham in any of the studies identified]); failure may have been because of the DTx being ineffective or because of inappropriately designed shams that produced strong therapeutic effects. Group 4 includes perhaps the ideal studies, in that the sham arm showed improvement from baseline, but the DTx arm showed significantly larger improvement (DTx++, Sh+), implying that the active components of the DTx were responsible for the greater efficacy.

## Results

### Overview

**Figure 1:**
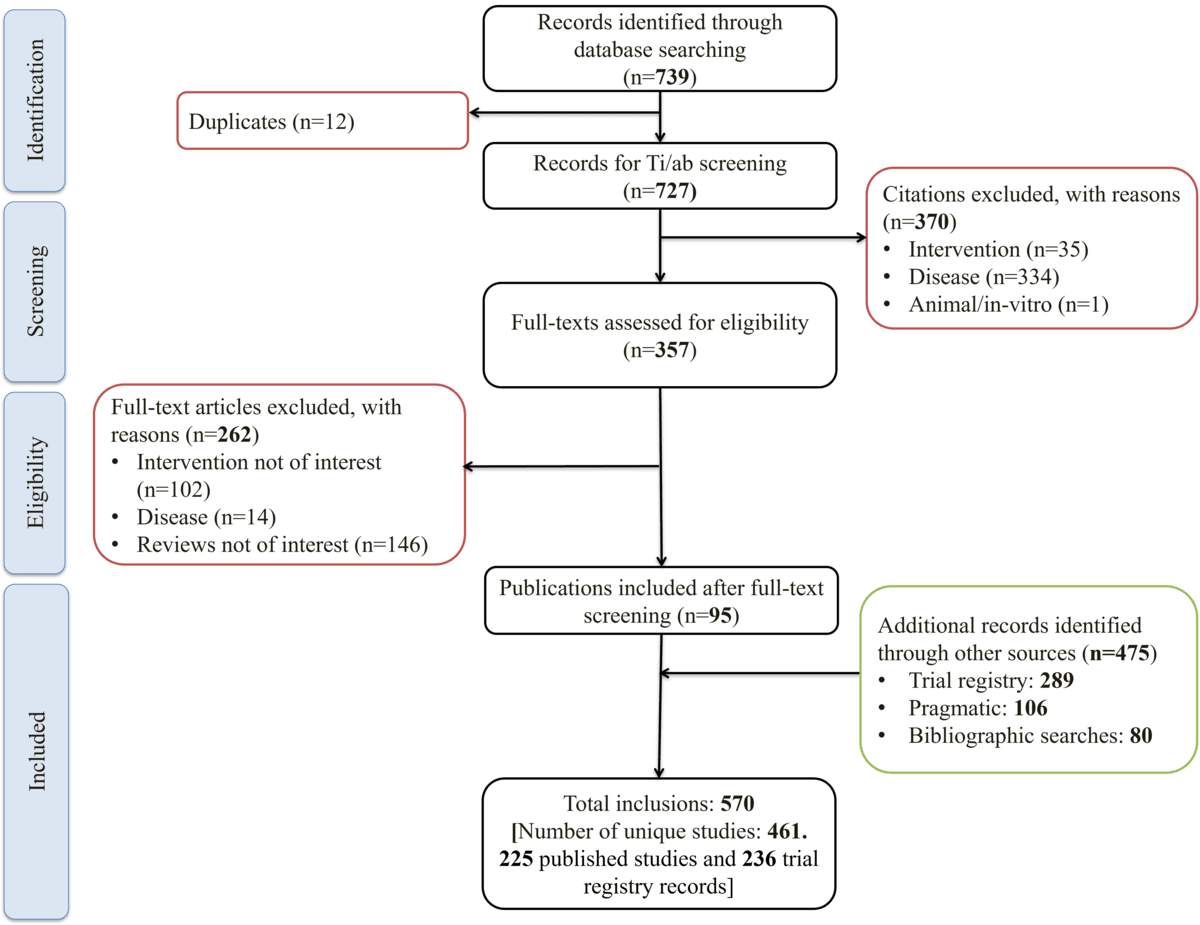
PRISMA (Preferred Reporting Items for Systematic Reviews and Meta-Analyses) flow diagram. The PRISMA diagram for studies included in the review is shown, with the number of records included or excluded at each step in parentheses. Intervention: studies not involving digital therapeutics (DTx) were excluded. Disease: studies involving DTx in nonneuroscience indications were excluded. Ti-ab: title-abstract.

As shown in the PRISMA (Preferred Reporting Items for Systematic Reviews and Meta-Analyses) flow diagram (Figure 1), database searches yielded 739 potentially relevant citations. Following title-abstract screening and a detailed full-text examination, 95 publications were identified (refer to Table S3 in Multimedia Appendix 1 for a list of studies excluded at this step). In addition, supplementary searches identified 186 publications and 289 registry records that met the eligibility criteria. With linking, this represented 461 unique studies (225 studies and 236 trial registry records).

Of the 225 studies (Table S4 in Multimedia Appendix 1), 203 (90.2%) were full publications (journal articles), 196 (87.1%) were published since 2015, a total of 147 (65.3%) were comparative studies, and 47 (20.8%) used a digital sham. Of the 236 registry records, 166 (70.3%) were comparative studies and 21 (8.9%) used a digital sham. The 313 comparative studies involved 213 unique DTx (Table S5 in Multimedia Appendix 1).

### Overview of Identified DTx

On the basis of the hardware and software components, we classified DTx as mobile device based (smartphone+software app), computer based (computer+software or video game), web based (link to access software accessible through computers or mobile devices), wearable (physical wearable devices), or XR based (headset+software or video game).

Of the 213 DTx, most were XR based (n=86, 40.4%) or mobile device based (n=56, 26.3%). The remaining DTx were computer based (33/213, 15.5%), web based (19/213, 8.9%), or wearables (19/213, 8.9%). Most DTx (126/213, 59.2%) were industry developed. DTx were most commonly developed for stroke (42/213, 19.7%), depression (32/213, 15%), anxiety (24/213, 11.3%), and substance use disorders (17/213, 8%). The DTx were most used for delivering CBT and behavioral therapy (69/213, 32.4%), physical rehabilitation (60/213, 28.2%), and cognitive training (41/213, 19.2%).

### Overview of Comparative Studies

Of the 313 comparative studies, most (n=303, 96.8 %) were randomized controlled trials. The comparator was a digital sham in 21.7% (68/313) of studies, an active control in 38% (119/313) of studies, a waitlist control in 11.5% (36/313) of studies, and treatment as usual or no intervention in 32.9% (103/313) of studies. Quantitative results (efficacy or safety) were available for 125 studies, of which DTx efficacy was successfully demonstrated in 103.

### Use of Digital Shams

Digital shams were comparators in 68 (21.7%) of the 313 studies. None of these was the sham validated for use as a placebo comparator; however, a few authors have cited prior research where the sham had demonstrated no clinical efficacy [47-49].

Digital shams were identical to the DTx in terms of hardware and duration of treatment, except in 1 study [50] where the digital sham sessions were shorter than the DTx sessions (refer to Table S6 in Multimedia Appendix 1 for the nature of digital shams and Table S7 in Multimedia Appendix 1 for the duration of DTx and digital shams).

Different approaches were used for *inactivating* the active components of digital shams (refer to Table S6 in Multimedia Appendix 1 for additional details). The most common approach (48/68, 71%) was to replace the active component with an inactive or neutral component, for example, replacing positive images with neutral images (therapeutic evaluative conditioning) [51]. In 9% (6/68) of the studies, the active component was simply removed; for example, the app showed only a timer instead of coping strategies (PEAR-004) [52]. In other studies (7/68, 10%), the active component was replaced by a different active component unrelated to the study outcomes; for example, a video game using CBT for attention-deficit/hyperactivity disorder (ADHD) was replaced with a game targeting cognitive domains not primarily associated with ADHD [53,54]. Finally, some studies (7/68, 10%) used digital shams that were less intense versions of the active treatment, for example, using a lower game difficulty level (Brain HQ) [55].

Excluding the studies and registry records for which only study protocols were available (25/68, 37%), 43 sham-controlled trials provided efficacy results. Of these 43, 32 (74%) provided efficacy data for digital shams, whereas the remaining 11 (26%) reported DTx efficacy without reporting the impact of digital shams on outcomes, that is, these studies reported information on the *difference between DTx and sham* over time but did not separately report quantitative information on changes in the sham arm alone (brief details in Table 1).

**Table 1:** Summary of 43 sham-controlled studies that provided efficacy results.

Study	Sample size, n	Length of the study (wk)	Treatment delivered	Indication	Blinding status	Name of DTx^a^	Type of DTx	Name of digital sham	Nature of digital sham	Comparison between DTx and digital sham	
										Dropout rate	Patient engagement or satisfaction
Rosa et al [56], 2017	6	12	Cognitive training	ADHD^b^	Single blind	ACTIVATE	Computer based	Placebo cognitive training	Inactive or neutral	Higher in DTx	NR^c^
Ghaemi et al [52], 2022	112	12	CBT^d^ or behavior therapy	Psychosis	Single blind	PEAR-004	Mobile devices based	PEAR-004 placebo app	Missing	Similar	NR
Cimenser et al [17], 2021	31	26	Others	Dementia	NR	GENUS (gamma entrainment using sensory stimuli) or synchronized 40-Hz gamma oscillation	Wearable	GENUS (gamma entrainment using sensory stimuli): sham settings	Inactive or neutral	Higher in digital sham	NR
Sosnowski et al [57], 2021	54	6	CBT or behavior therapy	ASD^e^	Double blind	Lookware	Computer based	Control video game	Inactive or neutral	Higher in digital sham	Similar
Kalmbach et al [47], 2020	91	7	CBT or behavior therapy	Insomnia	Single blind	Sleepio	Web based	Web-based sleep education or sleep hygiene education	Inactive or neutral	Similar	Similar
Kollins et al [53], 2020 (STARS-ADHD^f^)	348	4	Cognitive training	ADHD	Double blind	EndeavorRx (AKL-T01)	Mobile devices based	AKL-T09	Another active	Similar	NR
Yerys et al [58], 2019	19	4	Cognitive training	ASD	Single blind	Project-Evo	Mobile devices based	AKL-T09	Another active	Similar	Similar
Hancock et al [55], 2015	71	6	Cognitive training	MS^g^	Double blind	Brain HQ (InSight or BrainTwister visual n-back programs)	Computer based	Sham training (computerized cognitive training)	Lower intensity	Similar	NR
van der Molen et al [59], 2010	95	15	Cognitive training	Intellectual disability	Single blind	Odd Yellow training (Training A and B)	Computer based	Control training (with no demand on memory capacity)	Inactive or neutral	Similar	NR
Berman et al [60], 2020	89	6	CBT or behavior therapy	SUD^h^	Single blind	Telecoach	Mobile devices based	A web-based control app	Inactiveor neutral	Similar	NR
Teng et al [11], 2019	82	8.3	Others	Anxiety	Double blind	Home-delivered attentional bias modification	Mobile devices based	Placebo training	Inactive or neutral	Similar	NR
Batterham et al [48], 2018	194	15	CBT or behavior therapy	Anxiety	Single blind	FitMindKit	Web based	HealthWatch	Inactive or neutral	Similar	Higher with DTx
Davies et al [61], 2017	488	4	CBT or behavior therapy	SUD	Single blind	Drinks Meter	Mobile devices based	Imagination of information about alcohol misuse	Inactive or neutral	Similar	NR
Scholten et al [62], 2016	138	16	Others	Anxiety	Single blind	Dojo	Computer based	Rayman 2: The Great Escape	Inactive or neutral	Similar	NR
Schoneveld et al [63], 2016	134	13	CBT or behavior therapy	Anxiety	Single blind	MindLight	Computer based	Max and the Magic Marker	Inactive or neutral	Similar	Higher with digital sham
de Vries et al [64], 2015	121	12	Cognitive training	ASD	Double blind	*Braingame Brian*, an adaptive working memory training and cognitive flexibility	Computer based	Nonadaptive control training (mock-training)	Lower intensity	Similar	NR
Bikic et al [65], 2017	18	7	Cognitive training	ADHD	Double blind	Scientific Brain Training	Computer based	Placebo control arcade game (Tetris)	Another active	Higher in digital sham	NR
Chacko et al [66], 2014	85	8	Cognitive training	ADHD	Double blind	CWMT^i^	Computer based	CWMT placebo	Lower intensity	Similar	NR
Enock et al [12], 2014	429	12.6	Others	Anxiety	Double blind	Attention Bias Modification Training	Mobile devices based	Control training (no contingency training)	Inactive or neutral	Similar	NR
Bove et al [67], 2021 (DigCog)	44	14	Cognitive training	MS	Double blind	AKL-T03	Mobile devices based	AKL-T09	Another active	Higher in DTx	Similar
Garcia et al [68], 2021	188	8	CBT or behavior therapy	Pain	Double blind	EaseVRx headset with active intervention	Extended reality	EaseVRx headset without active intervention	Inactive or neutral	Similar	Similar
Pasula et al [69], 2020	252	NR	Others	Migraine	Double blind	NerivioMigra app (remote electrical neuromodulation device)	Wearable	NerivioMigra app (remote electrical neuromodulation device)—sham stimulation	Inactive or neutral	NR	NR
Wijnhoven et al [10], 2020	109	19	CBT or behavior therapy	Anxiety	NR	Mindlight	Computer based	Triple Town	Inactive or neutral	Similar	NR
Iacoviello et al [70], 2018	51	6	CBT or behavior therapy	Depression	Double blind	EFMT^j^	Computer based	EFMT—with neutral shapes	Inactive or neutral	Similar	NR
Parks et al [71], 2018	1051	8	CBT or behavior therapy	Anxiety	NR	Happify	Web based	Psychoeducation	Inactive or neutral	Higher in DTx	NR
Zwerenz et al [72], 2017	229	12	CBT or behavior therapy	Depression	Single blind	Deprexis	Web based	Web-based information about depression	Inactive or neutral	Similar	Higher with DTx
Deady et al [73], 2016	104	25.8	CBT or behavior therapy	Depression	Single blind	DEAL^k^ intervention	Web based	HealthWatch	Inactive or neutral	Similar	Higher with digital sham
Dovis et al [74], 2015	89	18	Cognitive training	ADHD	Double blind	Braingame Brian	Computer based	Braingame Brian on placebo mode	Lower intensity	Similar	NR
Espie et al [75], 2012	156	8	CBT or behavior therapy	Insomnia	Single blind	Sleepio	Web based	Imagery relief therapy	Inactive or neutral	Similar	NR
McEwen et al [76], 2014	59	7.3	Others	Stroke	Double blind	Virtual reality system (Interactive Rehabilitation Exercise software [IREX])	Extended reality	Control, virtual reality system (Interactive Rehabilitation Exercise software [IREX])	Inactive or neutral	Higher in digital sham	NR
Stasiak et al [77], 2014	34	10.3	CBT or behavior therapy	Depression	Double blind	The Journey	Computer based	Placebo program with psychoeducational content (computerized program with a brief psychoeducational content [CPE])	Inactive or neutral	Similar	NR
In et al [78], 2012	19	4	Others	Stroke	NR	Virtual reality reflection therapy program	Extended reality	Control (sham program)	Inactive or neutral	Similar	NR
Bentz et al [79], 2021	74	7	Others	Phobia	Single blind	Easy Heights	Extended reality	Google Street View app	Inactive or neutral	Higher in DTx	NR
Cheng et al [80], 2021 (Sleep to Prevent Evolving Affecting Disorders [SPREAD] trial)	1358	6	CBT or behavior therapy	Insomnia	Single blind	Sleepio	Web based	Web-based sleep education and sleep hygiene education	Inactive or neutral	Higher in DTx	NR
Espie et al [81], 2019 (Digital Insomnia Therapy to Assist Your Life as Well as Your Sleep [DIALS] study)	1448	24	CBT or behavior therapy	Insomnia	Single blind	Sleepio	Web based	Web-based sleep education and sleep hygiene education	Inactive or neutral	Higher in DTx	NR
Keefe et al [18], 2019 (STARS-MDD^l^)	74	6	Cognitive training	Depression	Double blind	AKL-T03	Mobile devices based	AKL-T09	Another active	NR	NR
Schenker et al [19,20], 2019	55	NR	Others	Migraine	Double blind	Relivion	Wearable	Relivion Device—sham stimulation	Lower intensity	NR	NR
Bucci et al [21], 2018	36	22	CBT or behavior therapy	Psychosis	Single blind	Actissist	Mobile devices based	ClinTouch app	Missing	Higher in digital sham	NR
Azevedo et al [82], 2017	52	NR	Others	Anxiety	Single blind	Doppel device	Wearable	Doppel device with active component (Doppel) turned off condition	Inactive or neutral	NR	NR
Dennis-Tiwary et al [83], 2017	33	4	Others	Anxiety	Double blind	Personal Zen: attention bias modification training	Mobile devices based	Placebo—attention training application	Inactive or neutral	NR	NR
Perry et al [84], 2017 (TriPoD)	540	77.4	CBT or behavior therapy	Depression	Double blind	SPARX-R (Smart, Positive, Active, Realistic, X-factor thoughts)	Web based	lifeSTYLE	Inactive or neutral	Higher in DTx	NR
Christensen et al [85], 2016 (GoodNight study)	1149	25.8	CBT or behavior therapy	Insomnia	Single blind	SHUTi	Web based	HealthWatch	Inactive or neutral	Higher in DTx	NR
Franklin et al [51], 2016	Study 1: 114, study 2: 131, and study 3: 163	4	CBT or behavior therapy	Depression	NR	TEC^m^	Mobile devices based	TEC with neutral (or blank) images	Inactive or neutral	Higher in digital sham	Similar

^a^DTx: digital therapeutics.

^b^ADHD: attention-deficit hyperactivity disorder.

^c^NR: not reported.

^d^CBT: cognitive behavioral therapy.

^e^ASD: autism spectrum disorder.

^f^STARS-ADHD: Software Treatment for Actively Reducing Severity of ADHD.

^g^MS: multiple sclerosis.

^h^SUD: substance use disorder.

^i^CWMT: Cogmed Working Memory Training.

^j^EFMT: Emotional Faces Memory Task training.

^k^DEAL: Depression-Alcohol Project.

^l^STARS-MDD: Software Treatments for Actively Reducing Severity of Cognitive Deficits in Major Depressive Disorder.

^m^TEC: therapeutic evaluative conditioning.

DTx or digital sham were considered to demonstrate efficacy if they showed statistically significant improvement in primary outcomes from baseline to follow-up. DTx was considered better than digital sham if there were statistically significant differences between the 2 groups at follow-up. For studies in which patients were not blinded per se but were not informed about whether the treatment they were receiving was active or control or in situations where the expectations from the 2 arms were equalized, they were considered to be blinded. For dropout rate and patient engagement or satisfaction rates, we considered a difference of >5% when reporting the DTx-sham comparison.

On the basis of the efficacy results of DTx and sham, the 32 studies were categorized as follows:

### Group 1: Studies Where Neither Sham Nor DTx Showed Improvement From Baseline (DTx–, Sh–; n=2)

In 2 studies, the DTx itself failed to show improvement from baseline. One study was underpowered, with data from only 6 patients, and the other was an unpublished study [52,86,87].

### Group 2: Studies Where DTx Showed Improvement From Baseline But Sham Did Not (DT+, Sh–; n=7)

All these studies involved CBT or behavioral therapy or cognitive training. The sample size ranged from 19 to 348 patients, and the study duration ranged from 4 to 7 weeks (except for 2 studies with longer duration). Three studies were double blind and 3 were single blind. In most studies (4/7, 57%), the active component of the DTx was replaced by an inactive or neutral component. Indications included autism spectrum disorder (n=2), ADHD, intellectual disability, dementia, MS, and insomnia (1 each).

In these papers, the authors discussed the results in the active treatment arms only in relation to the digital sham and did not specifically explain the results of the digital sham.

### Group 3: Studies Where Sham and DTx Showed Comparable Improvement From Baseline (DTx+, Sh+; n=10)

These included 4 studies involving CBT or behavioral therapy, 3 involving cognitive training, and 3 others involving attention bias modification training and emotion regulation training. The sample size ranged from 18 to 488 patients, and the study duration ranged from 4 to 16 weeks. Five studies were double blind and 5 were single blind. In most cases (7 studies), the active component of the DTx was replaced by an inactive or neutral component. The indications were autism spectrum disorder (1/10, 10%), ADHD (2/10, 20%), anxiety (5/10, 50%), and alcohol use disorder (2/10, 20%).

The key features of these studies and the reasons for their failure are summarized in Table 2. Only a few authors attributed study failure to an ineffective DTx (Table 2), noting deficiencies in control arms in previous studies that were remedied by an appropriately designed sham control in their study. In other cases, the authors felt that their DTx was probably effective but had not been implemented appropriately and needed to be made more engaging or intense. Most authors focused on the challenges with the sham control arm, speculating that the sham may have been too engaging or that the short-term distraction provided by the sham control played a large role. Some authors also proposed a more direct therapeutic effect of sham control on resilience, motivation, and cognition. Finally, some authors suggested that an adequately powered study may have succeeded in demonstrating the comparative efficacy of DTx.

**Table 2:** Reported reasons for failure of digital therapeutics (DTx) in studies that showed a positive impact of digital sham.

Study	Indication	DTx and sham	Potential reasons for study failure discussed by the study authors	Additional considerations
Berman et al [60], 2020	Problematic alcohol use	DTx: a web-based skills training smartphone app Sham: an app offering brief information and advice regarding problematic alcohol use	Highly motivated sample Less severely ill sample (those with hazardous use of alcohol were also included) High engagement with the app (analysis was done on 6-week completers)	Time spent on app was numerically higher with sham than DTx—31 min vs 20 min
Teng et al [11], 2019	Anxiety	DTx: ABM^a^; a pair of stimulus words provided—1 threatening and 1 neutral; the probe (E) replaced the neutral word Sham: identical to DTx, except that the probe randomly replaced the neutral or threatening word Waitlist control	The sessions themselves may have reduced anxiety as participants learned to inhibit unrelated stimuli and focus on the given task	Double-blind study Both DTx and sham were superior to the waitlist control
Batterham et al [48], 2018	Anxiety, depression, etc	DTx: brief targeted interventions across 18 modules—tailored and untailored versions used Sham: general health–related information; not associated with therapeutic reductions in depression	Sham may have been too active Underpowered study (note: n=194) Insufficient engagement with the intervention as it was fully automated Insufficient intensity of the intervention (intervention was broad and covering multiple domains) Less severely ill sample, therefore ceiling effect in symptom domains	Recommendations: A more comprehensive or more targeted version of the DTx Greater interactivity Measure coping and self-efficacy rather than only symptoms
Davies et al [61], 2017	Drinking issues	DTx 1: provided information about the consequences of excessive alcohol consumption and induced anticipated regret DTx 2: provided information on the consequences and provided normative information about others’ behavior and experiences Control 1 (sham): participants were asked to imagine that they are exposed to information about alcohol misuse, without receiving any alcohol information Control 2: no treatment	Underpowered study (note: n=402) Impact of completing measures of drinking and behaviors, that is, mere-measurement or question behavior effect Less severely ill sample (low-risk drinkers were included) Outcome data collected during a later part of the university semester, when the increased workload may have interfered with drinking behavior	Controls 1 and 2 were combined for analyses because the point estimates and CIs were very similar in both control groups
Scholten et al [62], 2016	Anxiety	DTx: video game that trained emotion regulation strategies and then challenged players to use the newly acquired strategies Sham: video game not explicitly designed to incorporate training components that target anxiety	Sham may have trained the same skills as DTx (video games are designed to urge players to work hard toward meaningful goals and to celebrate success) Duration of intervention may have been too long for the DTx (a few participants reported frustration with using the game) Applying intervention in group-based context may be unsuitable for adolescents who are anxious	Measures taken to ensure comparability between DTx and sham: Sham was chosen carefully—matched the type of attention required, and appeared to teach general skills that may be related to mental health outcomes (eg, perseverance) After treatment assignment, expectations were equalized, measured, and controlled for Recommendation: use a sham that is less emotionally stimulating and more focused on “cold” cognitive challenges
Schoneveld et al [63], 2016	Anxiety	DTx: game using multiple strategies for reducing anxiety (neurofeedback, exposure training, and ABMa) Sham: commercially successful game, not explicitly designed to incorporate evidence-based anxiety reduction techniques	Nonspecific factors Participants were primed with expectations of effective anxiety reduction High motivation Both games helped with resilience training, reappraisal skills, self-efficacy, and short-term distraction	Measures taken to ensure comparability between DTx and sham: Sham was chosen carefully—high-quality game that ensured engagement, age appropriate, and included an avatar who had to conquer fearful obstacles After treatment assignment, expectations were equalized
de Vries et al [64], 2015	ASD^b^	DTx: adaptive working memory training and an adaptive cognitive flexibility training Sham: training in which all tasks remained at a low, nonadaptive level	Underpowered study (note n=121) Sham may have trained EFs^c^ to some extent despite the EF demand being very low	Double-blind study Authors highlighted the difficulty in finding a sham that has no impact on EFs
Enock et al [12], 2014	Anxiety	DTx: ABM; a pair of faces shown—1 threatening and 1 neutral; the probe (E) replaced the neutral face Sham: identical to DTx, except that the probe randomly replaced the neutral or threatening face Waitlist control	Nonspecific factors (authors agreed that the contingency of probe placement was not the active component) Short-term distraction Confidence instilled by use of computerized treatment tool	Double-blind comparison of DTx and sham Recommendation: use several comparison conditions to isolate active components
Bikic et al [65], 2017	ADHD^d^	DTx: computerized cognitive exercises Sham: Tetris game	Underpowered study (n=18) Sham may have been more engaging than DTx Sham may have had some effect on cognition, for example, working memory	Double-blind study Participants found neither arm very interesting Recommendations: gamification of the DTx to improve engagement use cognitively nonchallenging sham; however, this can cause low engagement and attrition
Chacko et al [66], 2014	ADHD	DTx: CWMT^e^ Sham: CWMT with same games but low level and nonadaptive	Tighter control arm	Double-blind study Study was conducted with the aim of determining if the reported benefits with CWMT would still be seen if it was compared with a tighter control

^a^ABM: attentional bias modification.

^b^ASD: autism spectrum disorder.

^c^EF: executive function.

^d^ADHD: attention-deficit hyperactivity disorder.

^e^CWMT: Cogmed working memory training.

### Group 4: Studies Where Both Sham and DTx Showed Improvement From Baseline and DTx Was Significantly Better Than Sham (DTx++, Sh+; n=13)

This included 8 studies involving CBT or behavioral therapy, 2 involving cognitive training, and 3 involving others (balance training, rehabilitation, and stimulation). The sample size ranged from 19 to 252 patients (except 1 study involving 1051 patients), and the study duration ranged from 4 to 26 weeks. Seven studies were double blind, 3 were single blind, and 3 did not report the blinding status. In most cases (11/13, 85% studies), the active component of the DTx was replaced by an inactive or neutral component.

The indications were depression (4/11, 36%), anxiety (2/11, 18%), stroke (2/11, 18%), pain (1/11, 9%), migraine (1/11, 9%), MS (1/11, 9%), ADHD (1/11, 9%), and insomnia (1/11, 9%).

Notably, in both stroke studies, the patients were also receiving conventional stroke rehabilitation treatment in both arms. In addition, in the study by Wijnhoven et al [10], the 2 treatment arms differentiated on parent-rated anxiety but not on child-rated anxiety, whereas in the study by Deady et al [73], differences between DTx and sham arms did not persist at follow-up.

A summary of all studies based on the types of results discussed in Table 1, showing DTx type, blinding status, nature of sham, and other parameters, is presented in Figure S1 in Multimedia Appendix 1; high-level trends and their potential implications are covered in the Discussion section.

Seven studies had a sample size of <50 [50,51,56,58,65,77,78]. When the group-wise analysis was repeated without these small studies, the findings remained unchanged.

## Discussion

### Overview

We identified >300 controlled trials of DTx in neuroscience, of which only approximately 20% used a digital sham control. Trials involving XR technology were the most common and were mostly available in recent trial registry records. Stroke and depression or anxiety were the most common indications, whereas physical rehabilitation and CBT or behavioral therapy were the most common active treatments.

### Overview of Digital Shams

We categorized the studies into 4 groups (groups 1-4) based on the impact of DTx and sham on outcomes and used this framework to assess the impact of study design elements and evaluate author perspectives on the role of the digital sham.

We identified the following key issues:

- Sham controls were not validated before implementation.
- Sham treatments varied considerably, from being nearly identical to the DTx [55,64,66] to using different software programs altogether [53,65]. This was further compounded by the fact that the shams were often not described adequately, making it difficult to evaluate DTx-sham differences consistently, and there was insufficient information on elements that might impact outcomes such as the expected level of engagement or difficulty relative to the DTx.
- In 26% (11/43) of the studies, the impact of the sham on outcomes (ie, change in scores from baseline to end point) was not reported, with the studies reporting only DTx versus sham differences over time. This limited our ability to fully understand and explore the study results.
- Only 26% (11/43) of the studies reported the actual level of engagement and satisfaction with the sham (compared with DTx). For the remaining studies, it was difficult to discern how much of the DTx-sham differences were driven by differences in engagement between the 2 arms. A recent meta-analysis showed that higher engagement features in apps targeting depression and anxiety symptoms were associated with larger effect sizes. The authors discussed the use of persuasive system design and *gamification* to improve user engagement and, thereby, clinical outcomes with these apps. This implies that a rigorous comparison of 2 treatment arms would need them to be matched on engagement levels to avoid confounding. At the very least, the level of engagement across the 2 arms would need to be reported for transparency [88].

These issues limited the ability to compare the sham control arms across studies. For example, in studies that failed to differentiate DTx from sham, a few authors attributed study failure to DTx inefficacy [48,62,65], whereas most subjectively attributed it to the placebo effect or to the presence of active elements in the sham [11,63,66]. Underlying these challenges was the lack of consensus on what is considered a digital sham. In some cases, the control was not called *sham* or *placebo*, although it met the characteristics of a digital sham. Currently, DTx are well understood, and there exists a definition of DTx that is accepted in the field and has been formalized by the Digital Therapeutics Alliance [89]. However, there is no universal definition of digital sham (we had to build an operational definition for use in this review). We believe that this lack of consensus around what is a digital sham lies at the root of the subsequent issues seen in the reporting and interpretation of digital sham studies. Lutz et al [13] also identified similar issues in their review of controls for DTx, where they highlighted the inconsistencies in nomenclature and the fact that multiple terms such as sham, placebo control, digital control, and attention-matched placebo control have been used in the literature; these different terms may correspond to different definitions making interstudy comparisons difficult. The authors also noted inconsistencies in the reporting of the control arm in DTx studies and highlighted the need to report data on engagement levels with the control arm [13]. By analyzing the digital sham–related data systematically and across a larger data set, we were also able to quantify the extent of missing data in the literature: only 1 in 4 studies reported data on the level of engagement with sham, and only 1 in 4 studies reported the change from baseline in outcomes in the sham control arm.

### Recommendations on Digital Sham Design and Reporting

Textboxes 1 and 2 show parameters that we recommend should be included in the design and reporting of studies using digital shams. This information will help assess how well constructed the sham was, that is, whether it differed from the DTx in aspects other than the purported active components (*design*) and allow assessment of the sham versus the DTx in terms of engagement or motivation or adherence during the study, providing appropriate context for interpretation (*reporting*). These recommendations are DTx specific and may thus be combined with already existing generic frameworks to ensure adequate reporting of digital sham control arms [89]. Although these requirements may appear quite detailed, particularly when compared with designing and reporting sham controls in traditional pharmaceutical interventions, we believe these are needed and that these aspects would (or should) already have been considered by study teams at the time of designing the studies and selecting appropriate digital sham controls, specifically because of the characteristics that differentiates digital intervention from traditional biological interventions described in the Introduction section. Despite some differing opinions to incorporating robust placebo control requirements in DTx studies (based on concerns about potential false negatives or the belief that inadequate controls are acceptable in pilot studies so long as authors acknowledge those limitations), it is widely acknowledged that maintaining methodological rigor in these studies is paramount, especially for such a novel class of medical interventions where long-term effectiveness and value still has to be fully established. Merely demonstrating the benefits of DTx only compared with no treatment or a waitlist control through a full clinical development is insufficient for establishing a causal relationship with the active component of DTx, as it does not provide clarity on whether the effects are because of the active component or patient engagement or expectations or motivation. Therefore, it is necessary to use a robust sham control group that effectively addresses various factors, including retest effects, regression to the mean, social contact, and differential motivation and expectations across the study groups [63,90].

For studies that have been adequately reported (using the parameters in Textboxes 1 and 2), the study results can then be interpreted with more confidence using the algorithm shown in Table 3. For example, in a group 2 study (DTx+ Sh–), a *true positive* result (ie, the DTx is efficacious, and the DTx-sham difference can be fully attributed to the active components) would be more likely if the sham control arm was well constructed *and* showed equal or higher engagement or motivation or adherence compared with the DTx arm. In contrast, in a group 3 study (DTx+ Sh+), a *true negative* result (ie, the DTx is nonefficacious) would be more likely if the sham control arm was well constructed and showed equal or lower engagement or motivation or adherence compared with the DTx arm. Similarly, positive group 4 (DTx++ Sh+) studies would be considered *true positives*, unless there were considerable issues with the sham design. Note that this algorithm cannot be reliably applied across most of the current DTx literature because of inadequate information on the construction of the sham control arm, including the engagement levels seen in that arm during the studies.

**Study protocol: the following information about the sham arm should be determined a priori and reported as part of clinical trial protocols.**

**Validation**

- What is the scope of the study with respect to digital sham? Is it to identify and characterize active components of the digital therapeutics (DTx) or is it as a control arm to conclusively demonstrate DTx efficacy, that is, late phase validation of DTx?
- Whether the sham had been validated as a placebo control for this DTx in an earlier study

**Nature of difference between sham and DTx**

- What are the active components in the DTx?
- Whether the underlying software or technology in sham control was fundamentally different from that in the DTx
- Whether there were elements of the DTx that were *switched off* in the sham
- Whether the active component of the DTx was removed from the sham
- Whether the active component of the DTx was replaced with an inactive or neutral component
- Whether the sham was the same as the DTx but of lower intensity or difficulty compared with the DTx
- Whether the sham had adaptive levels (levels that changed based on users’ performance) that were comparable with those in the DTx
- Others

**Sham design for engagement and motivation**

- Whether the sham was designed to be comparable with the DTx in terms of engagement
- Whether the sham was designed to be as motivating and satisfying to users as the DTx

**Blinding and expectation matching**

- Whether the outcome assessments would be done in a blinded manner
- Whether the patients would be blinded. If not, whether the patients would be informed about which arm was active and which was control
- Whether expectations would be matched between the active and control arms, that is, whether the researchers would prime participants in both groups in an equal manner to expect improvement with their allocated intervention

**Session and treatment details**

- Number of sessions
- Duration of each session
- Duration of treatment

**Study results: following the conduct of the study, the following information should be reported in the study publications.**

**Blinding**

- Success of blinding (for blinded studies)

**Patient-reported experience with sham**

- Patient-reported difficulty levels with digital therapeutics (DTx) and sham
- Patient-reported satisfaction with DTx and sham
- Patient engagement levels with DTx and sham, based on, for example, time spent on the intervention, number of completed sessions, and level of difficulty reached

**Dropout**

- Dropout rates with DTx and sham

**Impact on outcomes**

- Magnitude of improvement from baseline, reported separately for DTx and sham arms

**Table 3:** High-level algorithm for interpretation of results of sham-controlled studies.

In the study, how did sham compare with the DTx^a^ in terms of engagement or motivation or adherence?			Was sham well constructed, that is, was it identical to DTx in all aspects except the purported active components?		
			Yes		No
**Negative results (DTx+ sham+)**					
	Sham>DTx (eg, sham more engaging)	May be false negative Need to analyze if there were additional active components of DTx that were shared with sham		May be false negative but complete interpretation is difficult	
	Sham≤DTx (eg, sham equally or less engaging)	Likely to be true negative		May be true negative but complete interpretation is difficult	
**Positive results (DTx+ sham−)**					
	Sham≥DTx (eg, sham equally or more engaging)	Likely to be true positive		May be true positive but complete interpretation is difficult	
	Sham<DTx (eg, sham less engaging)	May be false positive Need to analyze if there were additional active components on which DTx and sham differed		May be false positive but complete interpretation is difficult	

^a^DTx: digital therapeutics.

Given the heterogeneity of digital shams, all aspects listed in Textbox 2 will not always apply; therefore, we recommend reporting as much information as possible. In the future, we hope that a simple scale can be created to capture the required minimum information and perhaps derive a score or category classifying the sham in terms of comparability with the respective DTx.

### Recommendations on Study Design

In proof-of-concept studies, the sham should mimic the DTx as closely as possible, differing only in the putative active components. In this early phase of development, researchers may need to experiment with different sham control arms to identify and confirm the actual active components of the DTx. In addition, as the sham arm can reasonably be expected to have a substantial impact on efficacy on its own, the risk of a false-negative result will need to be addressed by a priori statistical planning, anticipating a reduced DTx versus sham difference, and ensuring that the sample size is large enough for adequate study power.

In confirmatory trials, although the sham control arm may be omitted in favor of a waitlist control or treatment-as-usual comparator, we recommend using validated shams to conclusively demonstrate the efficacy of DTx. Thus, a 3-arm study would be ideal, where the DTx arm would be compared with a digital sham as well as with a waitlist control or treatment-as-usual comparator.

On the basis of the trends in our analysis, we recommend that more attention be paid to sham controls in psychiatric indications such as anxiety and substance use disorder because of the high risk of the impact of shams on outcomes. In such studies, patients appeared to respond to aspects of games that were not considered to be an active component or to the fact that they were being interviewed. More attention also needs to be paid to studies involving cognitive training, which risks failure because of the high impact of shams on some cognitive outcomes. This was seen not only in studies that used a nonadaptive sham or a sham with a lower intensity of training but also with shams consisting of a simple game such as Tetris; in these studies, even the control interventions that were assumed to be inactive resulted in a significant effect on outcomes. In contrast, studies involving CBT or behavioral therapy were more consistently successful.

In addition, the sample size and study duration should be adequate, and studies should preferably be double blind. In our analysis, larger and longer studies and industry-sponsored trials (which were generally larger than academic trials and more likely to be double blind) were more likely to show positive results. Finally, the analysis could be stratified by engagement levels to estimate the dose-response relationship, and novel study approaches (eg, a sham run-in period) were used to identify placebo responders. Blinding in DTx studies can be challenging, particularly for DTx involving psychological and behavioral therapies. A digital sham with no active component may have low face validity because of which some participants may become unblind to the treatment assignment [62]. Challenges with blinding notwithstanding, there are sufficient advantages to blinding for us to recommend that this be implemented in DTx trials and that the success of the blinding be reported (Textbox 2).

In summary, there is no consensus definition of digital sham, and sham controls were used in only a small proportion (68/313, 21.7%) of the DTx studies. However, the role of digital shams remains underappreciated. For example, a recent review of evidence generation approaches in DTx noted the lack of high-quality evidence and discussed innovative strategies to meet the evidence requirements of the stakeholders; however, the challenges associated with digital sham were not discussed [91].

Our analysis shows that the use of sham control is, however, very important in the development of new DTx that are assessed using clinical trials, that is, DTx involving behavioral or training interventions that directly impact patient outcomes. Currently, there is a limited understanding of how to design an adequate sham control arm, and some sham-controlled trials may have failed for this reason. A lack of clarity regarding the active components of DTx can also result in inadequate sham controls; in many studies, key active components appeared to be shared between the DTx and sham [11,48,62,63,65]. We strongly recommend the use of sham-controlled studies in early research to identify DTx active ingredients, as this is a critical step in the optimization of the development of the intervention as well as in its validation. Given the central role of digital sham in generating robust controlled evidence, juxtaposed with our findings that demonstrate inadequacies in the use of sham controls in the literature, it is evident that implementing an adequately designed sham control is necessary to accurately evaluate the efficacy of DTx. Without appropriate sham-controlled data, the possibility of false-positive results cannot be ruled out. Finally, the sham control arm may need to adapt to changing DTx as the DTx evolves during clinical development; this could be an area of future investigation.

Although this review did not focus on the real-world applications of DTx, we noted that limited long-term and real-world data have been published on DTx efficacy. The study duration was ≤6 months in 82% (56/68) of the sham-controlled studies, and the transfer of acquired skills to long-term real-world outcomes was rarely assessed. Furthermore, assessing and separating the data into groups for analysis was not straightforward. In some studies, the identification of primary outcomes was not consistently reported, and the efficacy of DTx and sham interventions varied across different outcomes within these studies. Consequently, a nuanced evaluation is necessary to determine the overall effectiveness of the DTx and sham interventions. As noted earlier, another key challenge was the absence of a consensus definition for digital sham.

In addition to our focus on the definition and reporting of digital sham, there remains a need to appropriately define and implement other control strategies in DTx trials [62]. For instance, controls that vary from the DTx solely in terms of the intensity of the active component or in terms of attention and engagement also play a role in DTx development. Different control strategies and solutions are more or less appropriate at different stages of the clinical development of a DTx and based on the objectives of the clinical study. We advocate additional research in this area to establish standardized definitions for control arms in DTx trials more comprehensively.

### Gaps and Limitations

Screening, data extraction, data analysis, and categorization of the studies into groups were performed by a single reviewer. However, a quality check of all the steps was conducted to ensure the quality of this review.

In addition, despite our systematic approach, it is important to note that there might be DTx that we did not identify. This is because the field of DTx is vast and rapidly evolving. Furthermore, the line of demarcation between DTx and wellness apps is not always clearly defined. We distinguished between DTx and wellness apps based on the presence of well-developed software programs or algorithms. However, for a few therapeutic products, the underlying mechanism of action has not been adequately described in the available literature, and because of this, a definitive assessment could not be made. Finally, as the searches were completed in late 2021, the literature published from 2022 onward was not represented in this review.

### Conclusions

We believe that digital shams are critical to the clinical development of DTx. Sham-controlled studies should be routinely used in trials to help identify DTx active components (ie, early-phase studies) as well as to confirm the efficacy of DTx (ie, late-phase studies). The use of shams early in development will ensure that the appropriate sham control is used in later confirmatory trials. Overall, the inclusion of principles for sham design and deployment into best practice guidelines for clinical investigations of DTx will ensure the correct evaluation of benefits for patients and the health care system at large.

## Appendix

Multimedia Appendix 1 Additional tables and figures.

Multimedia Appendix 2 PRISMA Checklist.

## Abbreviations

ADHD: attention-deficit/hyperactivity disorder
CBT: cognitive behavioral therapy
DTx: digital therapeutics
MS: multiple sclerosis
PRISMA: Preferred Reporting Items for Systematic Reviews and Meta-Analyses
SPARX: Smart, Positive, Active, Realistic, X-factor
XR: extended reality

## Data Availability

The full data set will be made available upon request, after publication of this paper. Requests should be sent to DM.
