# Supplementary material for: Challenges With the Use of Digital Sham: Systematic Review and Recommendations
Source: J Med Internet Res. 2023 Oct 24;25:e44764. doi: 10.2196/44764 (PMC10630857; doi:10.2196/44764)
Supplement: Multimedia Appendix 1 [file jmir_v25i1e44764_app1.docx]

Challenges with the Use of Digital Sham: A Systematic Review and Recommendations Supplementary material

**Supplementary Table S1: Inclusion/exclusion criteria**

| **Domain** | **Inclusion/exclusion criteria** |
| --- | --- |
| Disease | - - - All indications related to neuroscience, neurodevelopment, neurodegeneration and psychiatric disorders were included     - Indications other than neuroscience were excluded* |
| Population | - - - Age-groups: All     - Ethnicity: Any |
| Intervention and comparator | - - - Studies assessing any DTx (with or without comparator) were included     - Products driven by high-quality software programs to prevent, manage, or treat a medical disorder or disease were considered as DTx     - Publications on smart devices (health systems that automatically capture patient physical parameters to proactively manage and deliver care) and connected drug delivery devices were excluded |
| Study design | - - - All clinical trials were included |
| Outcomes | - - - All outcomes were included |
| Publication timeframe | - - - 2010 onwards |
| Language | - - - English language articles only |
| Geography | - - - All |
| Other inclusion criteria | - - - Industry as well as academic research articles were included |
| DTx: Digital therapeutic. | |

*: Excluded studies included patients with non-neuroscience conditions such as metabolic disorders (diabetes, obesity), cancer, inflammatory bowel disease, asthma, cardiac diseases, COVID, arthritis, pregnancy etc.

**Supplementary Table S2: Embase/Medline Search Strategy**

| **No.** | **Query** | **Number of hits** | **Facet** |
| --- | --- | --- | --- |
| 1 | 'digital intervention'/exp OR 'digital health intervention'/exp | 30 | Digital Sham or Digital Therapeutics facet |
| 2 | 'digital sham':ab,ti OR 'digital placebo':ab,ti OR 'digital intervention':ab,ti OR 'digital interventions':ab,ti OR 'digital therapeutic':ab,ti OR 'digital therapeutics':ab,ti | 726 |  |
| 3 | #1 OR #2 | 744 | NA |
| 4 | #3 AND [english]/lim AND [2010-2021]/py | 739 | Final |

Searches were run on 10^th^ May 2021; NA: Not Applicable

**Supplementary Table S3: Studies excluded during full text screening**

| S no. | Title | Exclusion reasons |
| --- | --- | --- |
| 1 | Effort-optimized intervention model: Framework for building and analyzing digital interventions that require minimal effort for health-related gains | Review |
| 2 | Digital behavior change interventions for younger children with chronic health conditions: Systematic review | Review |
| 3 | Adults' preferences for behavior change techniques and engagement features in a mobile app to promote 24-hour movement behaviors: Cross-sectional survey study | Intervention not of interest |
| 4 | The economic case for digital interventions for eating disorders among United States college students | Intervention not of interest |
| 5 | An internet-delivered self-management programme for bipolar disorder in mental health services in Ireland: Results and learnings from a feasibility trial | Intervention not of interest |
| 6 | Clinician experiences of healthy lifestyle promotion and perceptions of digital interventions as complementary tools for lifestyle behavior change in primary care | Intervention not of interest |
| 7 | Workshop on the development and evaluation of digital therapeutics for health behavior change: Science, methods, and projects | Review |
| 8 | Evaluation framework for digital medicines in Europe | Review |
| 9 | Users' experiences of an online intervention for bipolar disorder: important lessons for design and evaluation | Intervention not of interest |
| 10 | Novel digital therapeutic improves visual acuity and encourages high adherence in amblyopic children | Disease not of interest |
| 11 | Digital therapeutics: Technology innovation to face general practice's challenges in 2020 | Review |
| 12 | Combining web-based gamification and physical nudges with an app (movemore) to promote walking breaks and reduce sedentary behavior of office workers: Field study | Disease not of interest |
| 13 | The need for artificial intelligence in digital therapeutics | Review |
| 14 | Digital and mobile technologies to promote physical health behavior change and provide psychological support for patients undergoing elective surgery: Meta-ethnography and systematic review | Review |
| 15 | Developing digital intervention games for mental disorders: A review | Review |
| 16 | Application of blended care as a mechanism of action in the construction of digital therapeutics | Review |
| 17 | Digital interventions for adolescent and young adult cancer survivors | Review |
| 18 | Using an analysis of behavior change to inform effective digital intervention design: How did the primit website change hand hygiene behavior across 8993 users? | Disease not of interest |
| 19 | Developing digital interventions for people living with serious mental illness: perspectives from three mhealth studies | Review |
| 20 | Potential benefits of incorporating peer-to-Peer interactions into digital interventions for psychotic disorders: A systematic review | Review |
| 21 | CLIMB: A mobile intervention to enhance social functioning in people with psychotic disorders: Results from a feasibility study | Review |
| 22 | Annual Research Review: Immersive virtual reality and digital applied gaming interventions for the treatment of mental health problems in children and young people: the need for rigorous treatment development and clinical evaluation | Review |
| 23 | Digital support interventions for the self-management of low back pain: A systematic review | Intervention not of interest |
| 24 | Effects of treatment length and chat-based counseling in a web-based intervention for cannabis users: Randomized factorial trial | Intervention not of interest |
| 25 | Developing a roadmap for digital health in cancer in Australia | Disease not of interest |
| 26 | Evaluation of reachout.com, an unstructured digital youth mental health intervention: Prospective cohort study | Intervention not of interest |
| 27 | Perceived helpfulness of a moderated online social therapy network for young people experiencing social anxiety | Intervention not of interest |
| 28 | Decision makers need an approach to determine digital therapeutic product quality, access, and appropriate use | Review |
| 29 | Study protocol for a systematic review of evidence for digital interventions for comorbid excessive drinking and depression in community-dwelling populations | Review |
| 30 | Using digital interventions to support individuals with alcohol use disorder and advanced liver disease: A bridge over troubled waters | Review |
| 31 | Predicting engagement with an online psychosocial intervention for psychosis: Exploring individual- and intervention-level predictors | Intervention not of interest |
| 32 | Role of kinematics assessment and multimodal sensorimotor training for motion deficits in breast cancer chemotherapy-induced polyneuropathy: A perspective on virtual reality avatars | Disease not of interest |
| 33 | Mobile intervention for depression benefits middle aged and older adults | Intervention not of interest |
| 34 | Mixed reality, full-body interactive experience to encourage social initiation for autism: Comparison with a control nondigital intervention | Intervention not of interest |
| 35 | Effect of adding a compassion-focused intervention on emotion, eating and weight outcomes in a commercial weight management programme | Intervention not of interest |
| 36 | Preparing nurses to be prescribers of digital therapeutics | Review |
| 37 | The impact of digital technology on psychological treatments and their dissemination | Review |
| 38 | Reported theory use by digital interventions for hazardous and harmful alcohol consumption, and association with effectiveness: Meta-regression | Review |
| 39 | Could digital therapeutics be a game changer in psychiatry? | Review |
| 40 | Challenges of and solutions for developing tailored video interventions that integrate multiple digital assets to promote engagement and improve health outcomes: tutorial | Disease not of interest |
| 41 | Identifying research priorities for digital technology in mental health care: results of the James Lind Alliance Priority Setting Partnership | Review |
| 42 | Profile of somryst prescription digital therapeutic for chronic insomnia: Overview of safety and efficacy | Review |
| 43 | Social isolation, mental health, and use of digital interventions in youth during the COVID-19 pandemic: A nationally representative survey | Intervention not of interest |
| 44 | Evidence synthesis of digital interventions to mitigate the negative impact of the COVID-19 pandemic on public mental health: Rapid meta-review | Review |
| 45 | AMCP Partnership Forum: Digital therapeutics—What are they and where do they fit in pharmacy and medical benefits? | Review |
| 46 | Effectiveness of a tailored, integrative Internet intervention (deprexis) for depression: Updated meta-analysis | Review |
| 47 | Theory integration for lifestyle behavior change in the digital age: An adaptive decision-making framework | Review |
| 48 | The state of mental digi-therapeutics: A systematic assessment of depression and anxiety apps available for Arabic speakers | Review |
| 49 | Digital interventions for mental disorders: Key features, efficacy, and potential for artificial intelligence applications | Review |
| 50 | The state of digital interventions for demand generation in low- and middle-income countries: considerations, emerging approaches, and research gaps | Review |
| 51 | Systematic review and critique of methods for economic evaluation of digital mental health interventions | Review |
| 52 | PMH18 Review and critic of methods for economic evaluation of digital mental health interventions | Review |
| 53 | Towards ehealth to support the health journey of headache patients: a scoping review | Review |
| 54 | Implementation and effectiveness of nonspecialist-delivered interventions for perinatal mental health in high-income countries: a systematic review and meta-analysis | Review |
| 55 | Influences on the uptake of and engagement with health and well-being smartphone apps: Systematic review | Review |
| 56 | Effects of a mobile and web app (thought spot) on mental health help-seeking among college and university students: Randomized controlled trial | Intervention not of interest |
| 57 | Pmd14 the evolution of regulatory frameworks of digital therapeutics (dtx) and implications for market access | Intervention not of interest |
| 58 | Past, current, and future willingness to engage with treatment targets: Applying user-centered design to inform the design of a mobile behavioral intervention | Intervention not of interest |
| 59 | From digital mental health interventions to digital “addiction”: Where the two fields converge | Review |
| 60 | Editorial: Digital interventions in mental health: current status and future directions | Review |
| 61 | PMU57 Where are we headed with digital therapeutics? A systematic review of the evidence | Review |
| 62 | Commentary: Let's get digital: a commentary on Halldorsson et al.'s call for more rigorous development and evaluation of immersive digital interventions for children and young people's mental health | Review |
| 63 | Digital extensions of clinical care in cancer: Stress management, adherence, and user-involvement | Review |
| 64 | A systematic review of treatments for alcohol-related cognitive impairment: lessons from the past and gaps for future interventions | Review |
| 65 | The transformation of health care for patients: Information and communication technology, digiceuticals, and digitally enabled care | Review |
| 66 | Effectiveness of a digital cognitive behavior therapy-guided self-help intervention for eating disorders in college women: a cluster randomized clinical trial | Intervention not of interest |
| 67 | Digital interventions for problematic cannabis users in non-clinical settings: findings from a systematic review and meta-analysis | Review |
| 68 | Prescription digital therapeutics: A new treatment modality for substance use disorders | Review |
| 69 | Preventing postpartum depression: a web-based intervention | Review |
| 70 | Psychosocial characteristics by weight loss and engagement in a digital intervention supporting self-management of weight | Intervention not of interest |
| 71 | Neuromodulation device with digital therapeutics, digital diagnostics, and artificial intelligence to treat neurology and neuropsychiatric conditions | Review |
| 72 | Induction of efficacy expectancies in an ambulatory smartphone-based digital placebo mental health intervention: randomized controlled trial | Intervention not of interest |
| 73 | Are digital interventions effective in reducing suicidal ideation and self-harm? A systematic review | Review |
| 74 | Digital interventions for PTSD symptoms in the general population: A review | Review |
| 75 | Are digitally delivered psychological interventions for depression the way forward? A review | Review |
| 76 | Digitally delivered psychological interventions for anxiety disorders: a comprehensive review | Review |
| 77 | Impact of a multicomponent digital therapeutic mobile app on medication adherence in patients with chronic conditions: Retrospective analysis | Intervention not of interest |
| 78 | Digital health interventions for children with ADHD | Review |
| 79 | Digital tools in the informed consent process: a systematic review | Review |
| 80 | Beyond pilotitis: Taking digital health interventions to the national level in China and Uganda | Review |
| 81 | Implementation of an online relatives' toolkit for psychosis or bipolar (impart study): iterative multiple case study to identify key factors impacting on staff uptake and use | Intervention not of interest |
| 82 | The effectiveness of prompts to promote engagement with digital interventions: a systematic review | Review |
| 83 | Integrated digital therapeutics and artificial intelligence in brain neuromodulation | Review |
| 84 | The current reimbursement landscape for digital health in the UK: how far are we from a centralised, national system? | Intervention not of interest |
| 85 | Gamifying CBT to deliver emotional health treatment to young people on smartphones | Review |
| 86 | Understand the mechanisms of behavior change in livewell: A smartphone intervention for bipolar disorder | Intervention not of interest |
| 87 | The role of digital medicine in autism spectrum disorder | Review |
| 88 | Virtual reality meets artificial intelligence: The emergence of advanced digital therapeutics and digital biomarkers | Review |
| 89 | Digital health interventions (DHI) for the treatment of attention deficit hyperactivity disorder (ADHD) in children - a comparative review of literature among various treatment and DHI | Review |
| 90 | The emergence of digital mental health in low-income and middle-income countries: A review of recent advances and implications for the treatment and prevention of mental disorders | Review |
| 91 | The evolution and future of mental health digital prevention for depression | Intervention not of interest |
| 92 | Digital healthcare for eating behavior | Intervention not of interest |
| 93 | Steering clear of driving after drinking: a tailored e-health intervention for reducing repeat offending and modifying alcohol use in a high-risk cohort | Intervention not of interest |
| 94 | Supporting antidepressant discontinuation: The development and optimisation of a digital intervention for patients in UK primary care using a theory, evidence and person-based approach | Review |
| 95 | Treatment of comorbid alcohol use disorders and depression with cognitive-behavioural therapy and motivational interviewing: a meta-analysis | Review |
| 96 | Apprehensions about excessive belief in digital therapeutics: points of concern excluding merits | Review |
| 97 | Digital interventions for autism spectrum disorder: A meta-analysis | Review |
| 98 | Use of the principles of design thinking to address limitations of digital mental health interventions for youth: viewpoint | Review |
| 99 | The therapeutic alliance in digital mental health interventions for serious mental illnesses: Narrative review | Review |
| 100 | Long-term maintenance of multitasking abilities following video game training in older adults | Disease not of interest |
| 101 | The peril of self-reported adherence in digital interventions: A brief example | Disease not of interest |
| 102 | Digital technology for health promotion: opportunities to address excess mortality in persons living with severe mental disorders | Review |
| 103 | Barriers to and facilitators of user engagement with digital mental health interventions: Systematic review | Review |
| 104 | Improving psychiatric care through integrated digital technologies | Review |
| 105 | The poem study: Testing the impact of a digital health platform in U.S. veterans with epilepsy | Intervention not of interest |
| 106 | Digital and technological opportunities in epilepsy: A future self-management ecosystem | Intervention not of interest |
| 107 | Research on key elements of digital therapeutics smartphone addiction | Disease not of interest |
| 108 | Game-based digital interventions for depression therapy: a systematic review and meta-analysis | Review |
| 109 | Perspectives of e-health interventions for treating and preventing eating disorders: descriptive study of perceived advantages and barriers, help-seeking intentions, and preferred functionality | Intervention not of interest |
| 110 | e-mental health interventions for the treatment and prevention of eating disorders: An updated systematic review and meta-analysis | Review |
| 111 | Can acceptance, mindfulness, and self-compassion be learned by smartphone apps? A systematic and meta-analytic review of randomized controlled trials | Review |
| 112 | Skills over pills? A clinical gastroenterologist’s primer in cognitive behavioral therapy for irritable bowel syndrome | Review |
| 113 | Closed-loop digital meditation for neurocognitive and behavioral development in adolescents with childhood neglect | Intervention not of interest |
| 114 | Plasticity of cognitive control in adolescents with adverse childhood experiences | Intervention not of interest |
| 115 | Use of a smartphone recovery tool for Latinos with co-occurring alcohol and other drug disorders and mental disorders | Intervention not of interest |
| 116 | Regulating digital therapeutics for mental health: Opportunities, challenges, and the essential role of psychologists | Review |
| 117 | Cope-support- A multi-component digital intervention for family carers for people affected by psychosis: Study protocol for a randomized controlled trial | Intervention not of interest |
| 118 | Digital interventions for screening and treating common mental disorders or symptoms of common mental illness in adults: Systematic review and meta-analysis | Review |
| 119 | Empowering the digital therapeutic relationship: virtual clinics for digital health interventions | Intervention not of interest |
| 120 | The digital placebo effect: Mobile mental health meets clinical psychiatry | Review |
| 121 | Commentary: A pilot digital intervention targeting loneliness in youth mental health | Review |
| 122 | Digital therapeutic self-management intervention in adolescents with migraine: feasibility and preliminary efficacy of “migraine manager” | Intervention not of interest |
| 123 | Predictors of overall and mental health-related internet use in adults with psychosis | Intervention not of interest |
| 124 | Predictors of engagement with a digital intervention for promoting personal recovery in persisting psychosis | Intervention not of interest |
| 125 | Developing a digital intervention for cancer survivors: an evidence-, theory- and person-based approach | Review |
| 126 | Developing digital interventions: A methodological guide | Review |
| 127 | PIH14 improving dementia economics through earlier cost-effective assessment | Intervention not of interest |
| 128 | Deriving patient personas with natural language processing for a digital therapeutic device | Intervention not of interest |
| 129 | That's just how I am': a qualitative interview study to identify factors influencing engagement with a digital intervention for tinnitus self-management | Intervention not of interest |
| 130 | Digital intervention with lifestyle coach support to target dietary and physical activity behaviors of adults with nonalcoholic fatty liver disease: Systematic development process of vitalise using intervention mapping | Disease not of interest |
| 131 | Digital health at fifteen: More human (more needed) | Intervention not of interest |
| 132 | Smartphone apps for the treatment of mental health conditions: status and considerations | Review |
| 133 | Toward clinical digital phenotyping: a timely opportunity to consider purpose, quality, and safety | Review |
| 134 | Use of theory in computer-based interventions to reduce alcohol use among adolescents and young adults | Review |
| 135 | The Pace of Technologic Change: Implications for Digital Health Behavior Intervention Research | Review |
| 136 | Clinic finds benefits from digital therapeutics | Intervention not of interest |
| 137 | Effectiveness of online and mobile telephone applications ('apps') for the self-management of suicidal ideation and self-harm: A systematic review and meta-analysis | Review |
| 138 | Brain-gut psychotherapy | Intervention not of interest |
| 139 | The application of digital health to the assessment and treatment of substance use disorders: The past, current, and future role of the National Drug Abuse Treatment Clinical Trials Network | Review |
| 140 | Information technology executives club in Rome: Position paper on information technology applications in medicine | Intervention not of interest |
| 141 | Novel advances in technology enhancements to mental health treatment for children and adolescents | Review |
| 142 | Information and communications technology-based interventions targeting patient empowerment: Framework development | Disease not of interest |
| 143 | Current use of artificial intelligence in the medtech industries | Intervention not of interest |
| 144 | Individual-level behavioural smoking cessation interventions tailored for disadvantaged socioeconomic position: a systematic review and meta-regression | Intervention not of interest |
| 145 | Therapist-supported online interventions for children and young people with tic disorders: Lessons learned from a randomized controlled trial and considerations for future practice | Intervention not of interest |
| 146 | Young people's experience of a long-term social media-based intervention for first-episode psychosis: Qualitative analysis | Intervention not of interest |
| 147 | Co-development of an interactive digital intervention to promote the well-being of people with intellectual disabilities | Intervention not of interest |
| 148 | The person-based approach to intervention development: application to digital health-related behavior change interventions | Review |
| 149 | Understanding and promoting effective engagement with digital behavior change interventions | Review |
| 150 | Reaching people with disabilities in underserved areas through digital interventions: systematic review | Review |
| 151 | Ensuring public trust in digital therapeutics: a pharmacopeial perspective | Review |
| 152 | The effectiveness of digital interventions for psychological well-being in the workplace: A systematic review protocol | Review |
| 153 | Commentary on Sundström et al. (2019): Digital interventions for alcohol problems-time for more research on blended therapy | Review |
| 154 | Digital therapeutics for tobacco use disorder in people with serious mental health conditions | Intervention not of interest |
| 155 | Participant perceptions of facilitators and barriers to adherence in a digital mental health intervention for a nonclinical cohort: Content analysis | Intervention not of interest |
| 156 | Feasibility and efficacy of the addition of heart rate variability biofeedback to a remote digital health intervention for depression | Intervention not of interest |
| 157 | Use of smartphones, computers and social media among people with SMI: opportunity for intervention | Intervention not of interest |
| 158 | Brief, web-based interventions to motivate smokers with schizophrenia: Randomized controlled trial | Intervention not of interest |
| 159 | Determinants of and barriers to adoption of digital therapeutics for mental health at scale in the NHS | Review |
| 160 | Sensing behavioral symptoms of mental health and delivering personalized interventions using mobile technologies | Intervention not of interest |
| 161 | Digital therapeutics: Emerging new therapy for neurologic deficits after stroke | Review |
| 162 | Digital interventions for people with co-occurring depression and problematic alcohol use: A systematic review and meta-analysis | Review |
| 163 | Barriers and facilitators to patient uptake and utilisation of digital interventions for the self-management of low back pain: A systematic review of qualitative studies | Review |
| 164 | Artificial intelligence, machine learning, and digital therapeutics in palliative care and hospice: The future of compassionate care or rise of the robots? (th363) | Intervention not of interest |
| 165 | Digital therapeutics for obesity and eating-related problems | Review |
| 166 | The many channels of screen media technology in ADHD: A paradigm for quantifying distinct risks and potential benefits | Review |
| 167 | E-INEBRIA special interest group roadmap for best practices for research on brief digital interventions for problematic alcohol and illicit drug use | Review |
| 168 | Online health information-seeking among older women with chronic illness: analysis of the women's health initiative | Intervention not of interest |
| 169 | Embodiment in virtual reality for the treatment of chronic low back pain: A case series | Intervention not of interest |
| 170 | Prior national drug abuse treatment clinical trials network (CTN) opioid use disorder trials as background and rationale for NIDA-CTN-0100 optimizing retention, duration and discontinuation strategies for opioid use disorder pharmacotherapy (RDD)"" | Intervention not of interest |
| 171 | Management of a high-performing mental health recovery research group | Intervention not of interest |
| 172 | The digital marshmallow test (DMT) diagnostic and monitoring mobile health app for impulsive behavior: development and validation study | Intervention not of interest |
| 173 | Suicide prevention using self-guided digital interventions: a systematic review and meta-analysis of randomised controlled trials | Review |
| 174 | 42.1 Youth engagement in digital intervention for anxiety: Lessons from initiation of the kids face fears pragmatic trial | Intervention not of interest |
| 175 | Strategies not accompanied by a mental health professional to address anxiety and depression in children and young people: a scoping review of range and a systematic review of effectiveness | Review |
| 176 | Clinical advances in obsessive-compulsive disorder: A position statement by the international college of obsessive-compulsive spectrum disorders | Intervention not of interest |
| 177 | Characteristics and challenges of the clinical pipeline of digital therapeutics | Review |
| 178 | A qualitative exploration of service user views about using digital health interventions for self-management in severe mental health problems | Review |
| 179 | Views and attitudes towards using the internet and mobile phones to receive support in severe mental health problems | Intervention not of interest |
| 180 | Using Artificial Intelligence to Predict Change in Depression and Anxiety Symptoms in a Digital Intervention: Evidence from a Transdiagnostic Randomized Controlled Trial | Intervention not of interest |
| 181 | Examining the initial usability, acceptability and feasibility of a digital mental health intervention for college students in India | Intervention not of interest |
| 182 | Emergence of digital biomarkers to predict and modify treatment efficacy: machine learning study | Intervention not of interest |
| 183 | The effect of brief digital interventions on attitudes to intellectual disability: Results from a pilot study | Intervention not of interest |
| 184 | 59.3 Digital interventions for homeless youth: problems and possibilities | Intervention not of interest |
| 185 | Internet-based psychological therapies: A qualitative study of National Health Service commissioners and managers views | Review |
| 186 | Promoting personal recovery in people with persisting psychotic disorders: Development and pilot study of a novel digital intervention | Intervention not of interest |
| 187 | Attitudes towards digital treatment for depression: A European stakeholder survey | Intervention not of interest |
| 188 | Digital cognitive-behavioral therapy in the treatment of adolescent depression: A randomized controlled trial | Intervention not of interest |
| 189 | A systematic literature review to assess the current evidence landscape for digital behaviour interventions in multiple sclerosis (MS) | Review |
| 190 | Digital therapeutics: An integral component of digital innovation in drug development | Review |
| 191 | Delivering information about medication assisted treatment to individuals who misuse opioids through a mobile app: A pilot study | Intervention not of interest |
| 192 | Slowmo, a digital therapy targeting reasoning in paranoia, versus treatment as usual in the treatment of people who fear harm from others: Study protocol for a randomised controlled trial | Intervention not of interest |
| 193 | Mobile software as a medical device (SAMD) for the treatment of epilepsy: Development of digital therapeutics comprising behavioral and music-based interventions for neurological disorders | Review |
| 194 | Implementing digital health for assessing and responding to clinical anxiety and depression (ADAPT) | Intervention not of interest |
| 195 | Digital interventions in adult mental healthcare settings: recent evidence and future directions | Review |
| 196 | Digital mental health apps and the therapeutic alliance: Initial review | Review |
| 197 | Qualitative evaluation of an explanatory model for somatoform symptoms (HERMES study): Acceptance and feasibility of a video-animated intervention | Intervention not of interest |
| 198 | Data-driven diagnostics and the potential of mobile artificial intelligence for digital therapeutic phenotyping in computational psychiatry | Review |
| 199 | Can-pain-a digital intervention to optimise cancer pain control in the community: development and feasibility testing | Review |
| 200 | Development of a novel digital app to optimise cancer pain management | Intervention not of interest |
| 201 | Implementation factors affecting the large-scale deployment of digital health and well-being technologies: A qualitative study of the initial phases of the 'Living-It-Up' programme | Intervention not of interest |
| 202 | A digital intervention for adolescent depression (moodhwb): Mixed methods feasibility evaluation | Intervention not of interest |
| 203 | Could adaptive research designs be useful in designing an effective ehealth intervention? A methodological analysis | Review |
| 204 | Digital therapeutics in the primary healthcare setting | Review |
| 205 | The development of balance retraining: an online intervention for dizziness in adults aged 50 years and older | Intervention not of interest |
| 206 | Harnessing psychology and technology to contribute to making health care a universal human right | Review |
| 207 | A digital intervention addressing alcohol use problems (the daybreak" program): Quasi-experimental randomized controlled trial" | Intervention not of interest |
| 208 | Exploring digital therapeutics: The next paradigm of modern health-care industry | Review |
| 209 | Toward game-based digital mental health interventions: player habits and preferences | Review |
| 210 | Mobile health technology interventions for suicide prevention: systematic review | Review |
| 211 | Digital technology for management of severe mental disorders in low-income and middle-income countries | Intervention not of interest |
| 212 | Access to evidence-based care for eating disorders during the COVID-19 crisis | Review |
| 213 | Psychological mechanisms in a digital intervention to improve physical activity: A multicentre randomized controlled trial | Intervention not of interest |
| 214 | Pilot randomized controlled trial of a novel smoking cessation app designed for individuals with co-occurring tobacco use disorder and serious mental illness | Intervention not of interest |
| 215 | Ideas (integrate, design, assess, and share): A framework and toolkit of strategies for the development of more effective digital interventions to change health behavior | Review |
| 216 | Addressing the research to practice gap through co-production & service user leadership in early psychosis intervention research | Intervention not of interest |
| 217 | Evaluating the role of digital intervention design in treatment outcomes and adherence to etherapy programs for eating disorders: A systematic review and meta-analysis | Review |
| 218 | Adherence to established treatment guidelines among unguided digital interventions for depression: Quality evaluation of 28 web-based programs and mobile apps | Review |
| 219 | Digital interventions in severe mental health problems: lessons from the Actissist development and trial | Review |
| 220 | Staff and service user perspectives of digital technology for early psychosis | Review |
| 221 | User-centered app adaptation of a low-intensity e-mental health intervention for Syrian refugees | Intervention not of interest |
| 222 | Effectiveness of theory-based digital self-management interventions for improving depression, anxiety, fatigue and self-efficacy in people with neurological disorders: A systematic review and meta-analysis | Intervention not of interest |
| 223 | Feasibility of biochemical verification in a web-based smoking cessation study | Intervention not of interest |
| 224 | AI in mental health | Review |
| 225 | The digital therapeutic alliance and human-computer interaction | Review |
| 226 | A systematic review of the effectiveness of digital interventions for illicit substance misuse harm reduction in third-level students | Review |
| 227 | Are computer-based treatment programs effective at reducing symptoms of substance misuse and mental health difficulties within adults? A systematic review | Review |
| 228 | Workshop on implementation science and digital therapeutics for behavioral health | Review |
| 229 | What works and what doesn’t work? A systematic review of digital mental health interventions for depression and anxiety in young people | Review |
| 230 | A new age intervention to support medication adherence | Review |
| 231 | Digital approaches to supporting parents with bipolar disorder: Lessons learned and future directions | Intervention not of interest |
| 232 | Exploring participants’ experiences of a web-based program for bulimia and binge eating disorder: Qualitative study | Intervention not of interest |
| 233 | Executive function in ADHD and related disorders: Multiple perspectives | Intervention not of interest |
| 234 | Affirm online: Utilising an affirmative cognitive–behavioural digital intervention to improve mental health, access, and engagement among LGBTQA+ youth and young adults | Intervention not of interest |
| 235 | Digital health developments and drawbacks: a review and analysis of top-returned apps for bipolar disorder | Review |
| 236 | A systematic review of technology-based prevention and treatment interventions for perinatal depression and anxiety in Latina and African American women | Review |
| 237 | Digital therapeutics as a medical benefit: Emergence of published policies by managed care organizations | Review |
| 238 | Engaging children and young people in digital mental health interventions: Systematic review of modes of delivery, facilitators, and barriers | Review |
| 239 | The emerging world of digital therapeutics | Review |
| 240 | Developing and evaluating digital interventions to promote behavior change in health and health care: Recommendations resulting from an international workshop | Review |
| 241 | Debate: Supporting the mental health of school students in the COVID-19 pandemic in New Zealand – a digital ecosystem approach | Review |
| 242 | 6.3 Beyond apps: integrating technology in practice | Intervention not of interest |
| 243 | Feasibility and effects of digital interventions to support people in recovery from substance use disorders: Systematic review | Review |
| 244 | Leveraging the social network for treatment of social anxiety: Pilot study of a youth-specific digital intervention with a focus on engagement of young men | Intervention not of interest |
| 245 | The global experience of digital health interventions in COVID-19 management | Review |
| 246 | Easipro3-enhancing satisfaction with prostate cancer treatment decision with the mobile health program prostana: A multicenter randomized controlled trial | Disease not of interest |
| 247 | Digital health implications for clinical pharmacists services: A primer on the current landscape and future concerns | Disease not of interest |
| 248 | Digital therapeutics in Parkinson’s disease: practical applications and future potential | Review |
| 249 | Beyond the trial: Systematic review of real-world uptake and engagement with digital self-help interventions for depression, low mood, or anxiety | Review |
| 250 | An evaluation of a digital pain management programme: clinical effectiveness and cost savings | Disease not of interest |
| 251 | Harnessing CURATE.AI as a digital therapeutics platform by identifying N-of-1 learning trajectory profiles | Review |
| 252 | Single-Session digital intervention for adolescent depression, anxiety, and well-being: Outcomes of a randomized controlled trial with Kenyan adolescents | Intervention not of interest |
| 253 | How digital therapeutics" differ from traditional health and wellness apps" | Intervention not of interest |
| 254 | Paradigm shift toward digital neuropsychology and high-dimensional neuropsychological assessments: Review | Intervention not of interest |
| 255 | The future of obsessive-compulsive spectrum disorders: A research perspective | Intervention not of interest |
| 256 | Innovative access pathways for digital healthcare solutions: Learnings from a European analogue analysis for Asian HTA bodies | Intervention not of interest |
| 257 | A conceptual economic model for digital interventions in serious mental illness | Intervention not of interest |
| 258 | On the use of digital technologies to reduce the public health impacts of cannabis legalization in Canada | Review |
| 259 | Digital interventions and attention deficit hyperactivity disorder | Review |
| 260 | An overview of digital health in the transition of pediatric to adult epilepsy care | Review |
| 261 | The promotion of eating behaviour change through digital interventions | Intervention not of interest |
| 262 | Regulation and accreditation of addictive behaviour apps - navigating the landscape | Review |

**Supplementary Table S4: Summary of characteristics of included studies**

| **Parameters** | **Details** | **Number of studies** | |
| --- | --- | --- | --- |
|  |  | **Studies**  **(N=225)** | **Registry studies**  **(N=236)** |
| **Type of publication** | Journal articles | 203 | NA |
|  | Conference abstracts | 22 | NA |
| **No. of studies by year of publication** | 2021 | 23 | NA |
|  | 2020 | 55 | NA |
|  | 2019 | 27 | NA |
|  | 2018 | 31 | NA |
|  | 2017 | 28 | NA |
|  | 2016 | 20 | NA |
|  | 2015 | 12 | NA |
|  | 2010 - 2014 | 29 | NA |
| **Comparative/non-comparative studies** | Comparative | 147 | 166 |
|  | Non-comparative | 78 | 70 |
| **Digital sham comparator*** | Yes | 47 | 21 |

NA: Not applicable.

*The comparator in the study was considered as digital sham if authors clearly stated the comparator to be sham/placebo (n=58) or when authors did not call the comparator a sham/placebo but the description of the comparator was in keeping with that of a sham control (n=10).

Supplementary Table S5: List of identified DTx

| Name of DTx | DTx developer | Indication | Approval status^*^ | Countries where approved |
| --- | --- | --- | --- | --- |
| Mobile device-based | | | | |
| 0-anxiety | NR | Anxiety disorders | NA | NA |
| A4i – App4Independance | App4Independence | Schizophrenia | In development | NA |
| Actissist | University of Manchester (UK) | Psychosis | NA | NA |
| AKL-T01 (EndeavorRx) | Akili Interactive Labs, Inc. | ADHD, depression, ASD, cognitive dysfunction | Approved | USA |
| AKL-T03 | Akili Interactive Labs, Inc. | Multiple sclerosis, depression | In development | NA |
| Am Mindfulness app (AmDTx) | Mobio Interactive | Stress- negative physical and psychosocial symptoms in cancer patients | Launched | NA |
| Attention Bias Modification Training | NR | Anxiety (social anxiety) | NA | NA |
| Attune | Blue Note Therapeutics | Anxiety and depression | In development | NA |
| BioBase | BioBeats Group Ltd, London | Stress, anxiety | Launched | NA |
| Cerena | Blue Note Therapeutics | Anxiety and depression | In development | NA |
| Chatbot- CBT (Todaki) | Gangnam Severance Hospital, FNI, and Selvas AI | ADHD, depressive disorder, bipolar disorder, anxiety disorder | No information available | NA |
| Constant Therapy (cognitive rehabilitation application) | Constant Therapy Health | Alzheimer's disease, aphasia | In development | NA |
| Continuing Care | COG Analytics | Substance use disorders | In development | NA |
| CT-101-M (Clickotine) | Click therapeutics | Smoking cessation | Approved | USA |
| Daylight | Big Health Ltd. | Anxiety | Approved | USA |
| Dcombat | Sponsor: Babes-Bolyai University Collaborator: Norwegian University of Science and Technology and CheckWare AS | Depression | Launched | NA |
| Drink less app | University College London | Substance use disorder (alcohol) | NA | NA |
| Drinks Meter | Professor Adam Winstock (UK) | Substance use disorder (alcohol) | NA | NA |
| eSleep | NR | Insomnia | NA | NA |
| Feel Stress Free | Thrive Therapeutic Software Limited | Anxiety and depression | Launched | NA |
| Find me app | University of Edinburgh | Autism | NA | NA |
| FOCUS app | UW BRiTE center - University of Washington | Schizophrenia, schizoaffective disorder, bipolar disorder, or major depressive disorder | NA | NA |
| Gotcha! Therapy application | University College, London | Dementia | NA | NA |
| HeadGear | Black Dog institute | Depression | NA | NA |
| Headspace mindfulness meditation app | Headspace | Insomnia, depression, anxiety, substance use disorder (alcohol) | Launched | NA |
| Healthy Paths and Healthy Mind app | LifeGuide software, University of Southampton | QoL in cancer survivors, anxiety | NA | NA |
| HeartMath + Inner balance sensor | HeartMath company | Migraine | Launched | NA |
| Home-delivered attentional bias modification (HD-ABM) | NR | Anxiety | NA | NA |
| iCanQuit (SmartQuit) | Fred Hutchinson Cancer Research Center in conjunction with 2Morrow Inc | Smoking cessation | Launched | NA |
| Intellicare app | Adaptive Health, Inc | Depression | Launched | NA |
| iTALKbetter (reactive) | University College, London | Stroke | NA | NA |
| Kaia Back Pain App | Kaia Health Software | Back pain | Approved | USA, EU |
| Limbix Spark | Limbix Health, Inc. | Depression | Launched (temporary access by FDA in Covid 19) | NA |
| Location-Based Monitoring and Intervention for Alcohol Use Disorders (LBMI-A) | University of Alaska | Substance use disorder (alcohol) | NA | NA |
| Marble Maze Classic® | Microsoft | Down syndrome | Launched | NA |
| Meru Health Ascend app | Meru Health Inc | Depression | In development | NA |
| MindMate + Neurotrack | MindMate Inc. | Alzheimer's disease | Launched | NA |
| MIRAI | Click Therapeutics, Inc. and Otsuka pharmaceutical development | Major depression | In development | NA |
| Pear-002a | Pear Therapeutics | Substance use disorder (opioids) | In development | NA |
| PEAR-004 | Pear Therapeutics | Schizophrenia | Launched (temporary access by FDA in Covid 19) | NA |
| Personal Zen: Attention bias modification training (ABMT) | NR | Stress and anxiety during pregnancy | NA | NA |
| Personalized Augmented Cognitive Training (PACT) | NR | Traumatic brain injury | NA | NA |
| Point OutWords | Com DEALL Trust in Bangalore, India | Verbal skills in autism | NA | NA |
| PRIME app | University of California-San Francisco | Schizophrenia | NA | NA |
| Quit Genius | Digital Therapeutics Inc | Smoking cessation | Launched | NA |
| reSET and reSET-O | Pear Therapeutics | Substance use disorder (opioids) | Approved | USA |
| reSET-O new (PEAR-008) | Pear Therapeutics | Substance use disorder (opioids) | In development | NA |
| ReZone | University College London | Stress | NA | NA |
| So-Lo-Mo | NR (So Lo Mo is a part of SmokeFreeBrain http://smokefreebrain.eu/) | Smoking cessation | NA | NA |
| Stressproffen | Oslo University Hospital | Stress management in cancer | NA | NA |
| SuperBetter | SuperBetter, LLC (now owned by Cherry Street Innovation) | Depression | Launched | NA |
| TeleCoach | NR | Substance Use Disorder (alcohol) | NA | NA |
| The Therapeutic Outcome By You (TOBY) | NR | ASD | NA | NA |
| Therapeutic Evaluative Conditioning (TEC) | tec-tec, LLC. | Self-harm- self injurious thoughts and behaviors | Launched | NA |
| WeChat app - eCBTI | Zeen health (Beijing) Technology Co., Ltd | Insomnia | No information available | NA |
| Xploro (DTx platform) | Xploro Limited | Anxiety- procedural knowledge, procedural anxiety, and reported experiences for a planned procedure) | Launched | NA |
| Web-based | | | | |
| Cognifit | CogniFit | Schizophrenia, brain injury | Launched | NA |
| DEAL Project (DEpression ALcohol) intervention | National Drug and Alcohol Research Centre, Australia | Depression and substance use disorder (alcohol) | NA | NA |
| Deprexis | Gaia AG | Depression | Approved | USA |
| Digital cognitive-behavioral therapy for insomnia (dCBTI) | NR | Insomnia | NA | NA |
| EMYNA | Gaia AG | Depression (epilepsy-specific) | Launched | NA |
| eSMART-MH | Case Western Reserve University | Depression | NA | NA |
| FitMindKit | AFFIRM, The Australian Foundation for Mental Health Research, and the John James Memorial Foundation | Bipolar disorder, depression, anxiety, substance use and suicidality | NA | NA |
| Growth mindset intervention | Fred Hutchinson Cancer Research Center and 2Morrow Inc | Smoking cessation | Launched | NA |
| Happify | Happify Health | Loneliness; depression and anxiety | In development | NA |
| LEAP | NR | Prevention of suicide (perceived burdensomeness) | NA | NA |
| Living with deadly thoughts (Leven onder Controle) | NR | Suicidal thoughts | NA | NA |
| MoodHelper | Kaiser Permanente Center for Health Research | Depression | NA | NA |
| PEAR-003b/ Somryst/ SHUTi | Pear Therapeutics | Insomnia, substance use disorders | Approved | USA |
| Plan-It Commander | Ranj game studio | ADHD | Launched | NA |
| RestDep | University of Bergen | Major depression | NA | NA |
| SelFIT | University of Bern | Stress- adjustment problems after an accident | NA | NA |
| SilverCloud Health intervention | Silvercloud health | Stress, depression, eating disorders and anxiety disorder | Launched | NA |
| Sleepio | Big Health Ltd. | Insomnia, depression, anxiety, psychotic disorders | Approved | USA |
| Velibra | GAIA | Anxiety | In development | NA |
| Extended reality | | | | |
| 3MDR With Eye Movement Component (EM+) | NR | PTSD | NA | NA |
| Augmented Reality Multi-Modal Training | NR | Parkinson's disease | NA | NA |
| Automated VR: Now I can do heights | Oxford VR | Fear of heights | Launched | NA |
| Balance Training | NR | Cognitive dysfunction, peripheral neuropathy | NA | NA |
| Bimodal VR-Stroop (ClinicaVR: Apartment Stroop) | NR | Acquired brain injury | NA | NA |
| BrightBrainer Virtual Reality (BBVR) Rehabilitation System | Bright Cloud International Corp | Traumatic brain injury | Approved | USA |
| Broom X, Virtual Reality | BroomX Technologies | Dementia | Launched | NA |
| Commercially-Available Interactive Video Games ("Wii Sports", "Wii Fit", EyeToy "Play 2" and "Kinetic") | Nintendo and Sony | Stroke | Launched | NA |
| Computer Assisted Rehabilitation Environment (CAREN) | MOTEK BV | Panic disorder, stroke | Approved | EU |
| Dynamic Interactive Social Cognition Training in Virtual Reality (DiSCoVR) | CleVR BV | Psychotic disorders | No information available | NA |
| EaseVRx | AppliedVR Inc. | Chronic low-back pain | Approved | US |
| Easy Heights | University of Basel | Fear of heights | NA | NA |
| gameChange VR treatment | Oxford VR | Psychosis | In development | NA |
| GRAIL | Motek Medical B.V. | Cerebral palsy, brain injury | Approved | EU |
| GripAble device - mobile rehabilitation system | GripAble HEALTHCARE | Stroke | Launched | NA |
| Immersion VR via HTC Vive | NR | Subacute stroke | NA | NA |
| Immersive virtual therapy | NR | Multiple sclerosis | NA | NA |
| Immersive virtual therapy | NR | Stroke | NA | NA |
| Immersive Virtual-based vestibular rehabilitation | NR | Multiple sclerosis | NA | NA |
| Intendo (Functional Brain Trainer VR) | Intendu Technologies | Bipolar disorder, depression | Launched | NA |
| Jintronix Rehabilitation Software - motion capture technology | Jintronix | Stroke | Approved | USA |
| Kinect based balance training | NR | Parkinson's disease | NA | NA |
| Kinect based Rapid Movement Therapy (Balance training) | NR | Stroke | NA | NA |
| Kinect for Xbox Virtual Reality Games - Pilates exercises | Microsoft | Multiple sclerosis | Launched | NA |
| Kinect for Xbox Virtual Reality Games (Adventure games) | Microsoft | Parkinson's disease | Launched | NA |
| Kinect for Xbox Virtual Reality Games (four games of X-Box Kinect system) | Microsoft | Parkinson's disease | Launched | NA |
| Kinect for Xbox Virtual Reality Games (Kinect Adventures and Kinect Sports) | Microsoft | Chronic ambulatory traumatic brain injury | Launched | NA |
| Kinect for Xbox Virtual Reality Games (Kinect games Adventures® Dance®) | Microsoft | Parkinson's disease | Launched | NA |
| Kinect for Xbox Virtual Reality Games (Kinect Sports I ®, Kinect Sport II ®, Kinect Joy Ride ® and Kinect Adventures ®.) | Microsoft | Stroke | Launched | NA |
| Kinect for Xbox Virtual Reality Games (Kinect Sports I^TM^, Kinect Joy Ride^TM^ and Kinect Disneyland Adventures^TM^) | Microsoft | Cerebral palsy | Launched | NA |
| Kinect for Xbox Virtual Reality Games (Kinect sports rivals) | Microsoft | Cerebral palsy | Launched | NA |
| Kinect for Xbox Virtual Reality Games | Microsoft | Stroke | Launched | NA |
| Kinect Xbox Boxing | Microsoft | Stroke | Launched | NA |
| Kinect-based Rehabilitation | NR | Stroke | NA | NA |
| Maze game software | NR | Parkinson's disease | NA | NA |
| Microsoft Kinect Games-Carnival Games | Microsoft | Cerebral palsy | Launched | NA |
| Mystic Isle | University of Southern California | Traumatic brain injury | NA | NA |
| Nature-VR | NR | Cognitive dysfunction in heart failure | NA | NA |
| NeuroDRIVE: VR Driving | NR | Traumatic brain injury | NA | NA |
| Nintendo Switch Joy-Con | Nintendo | Multiple sclerosis | Launched | NA |
| Nintendo Wii - balance and aerobic exercises | Nintendo | Alzheimer's disease | Launched | NA |
| Nintendo Wii - Balance Bubble Plus and Tennis, Rhythm Parade and Boxing games | Nintendo | Stroke | Launched | NA |
| Nintendo Wii - static balance, dynamic balance and walking | Nintendo | Parkinson's disease | Launched | NA |
| Nintendo Wii - Wii sports and Wii fit boxing exercises | Nintendo | Parkinson's disease | Launched | NA |
| Nintendo Wii (8 Wii gaming sessions) | Nintendo | Stroke | Launched | NA |
| Nintendo Wii Fit - 8 balance games | Nintendo | Stroke | Launched | NA |
| Nintendo Wii Fit plus - soccer heading, ski slalom and table tilt | Nintendo | Multiple sclerosis | Launched | NA |
| Nirvana | BTS Bioengineering | Stroke | Launched | NA |
| Oculus Rift (HMD VR + Training) | NR | Stroke | NA | NA |
| Oculus Rift and Leap motion | NR | Stroke | NA | NA |
| PlayStation®VR | Sony | Stroke | Launched | NA |
| Reh@City | NR | Stroke, substance use disorder (alcohol) | NA | NA |
| REMINISCENCES, personalized video: virtual reality session | NR | Bipolar disorder, depression | NA | NA |
| Self-training using video-games | NR | Stroke | NA | NA |
| Snow world | University of Washington HITLab in collaboration with Harborview Burn Center | Pain- painful wound care procedures (decrease in opioid use during procedure) | NA | NA |
| Systemic Lisbon Battery - Virtual reality cognitive training- | Lusophone University of Humanities and Technologies | Substance use disorder (alcohol) | NA | NA |
| Treadmill + VR | NR | Parkinson's disease | NA | NA |
| Tripp | TRIPPP Inc. | Stress- perioperative stress reduction in cancer patients | Launched | NA |
| Virtual reality | NR | Stroke | NA | NA |
| Virtual Reality | NR | Insomnia- reducing awakenings and increasing sleep efficiency in patients suffering from diabetic polyneuropathy | NA | NA |
| Virtual Reality (game training) | NR | Stroke | NA | NA |
| Virtual Reality (Games) | NR | Stroke | NA | NA |
| Virtual Reality aggression prevention training (VRAPT) | NR | Aggression prevention | NA | NA |
| Virtual Reality Attention Training (VRAT) | NR | Stroke | NA | NA |
| Virtual Reality Intervention | NR | Multiple sclerosis | NA | NA |
| Virtual Reality Intervention | NR | Stroke | NA | NA |
| Virtual Reality program | NR | Stroke | NA | NA |
| Virtual reality reflection therapy program | NR | Chronic stroke | NA | NA |
| Virtual Reality Software for Chronic Pain (VIRPI) | Orion Corporation, Orion Pharma | Chronic low-back pain | In development | NA |
| Virtual Reality system (IREX) | GestureTek | Stroke | Launched | NA |
| Virtual Reality Therapy | NR | Panic disorder - with agoraphobia | NA | NA |
| Virtual Reality Therapy using Kinect- motion sensor + mCIMT | NR | Cerebral palsy | NA | NA |
| Virtual Reality Training Session- Game-Based Rehabilitation | NR | Stroke | NA | NA |
| Virtual reality-based cognitive training | NR | Stroke | NA | NA |
| Virtual task using communication device systems (Kinect®, Leap Motion Controller® or touchscreen) | Information Systems Team at the University of Sao Paulo | Amyotrophic lateral sclerosis | NA | NA |
| VR intervention via HTC Vive | NR | Aphasia | NA | NA |
| VR with Kinect | NR | Cerebral palsy | NA | NA |
| VR-CBT | NR | Psychotic disorders | NA | NA |
| VR-GAIME (Virtual Reality Game for Aggressive Impulse Management) | NR | Aggression regulation problems | NA | NA |
| VR-training | NR | Parkinson's disease | NA | NA |
| Wii Fit - balance games and Wii Sports -bowling, golf and tennis games | Nintendo | Stroke | Launched | NA |
| Wii Sport Games | Nintendo | Cerebral palsy | Launched | NA |
| Wii virtual reality games | Nintendo | Cerebral palsy | Launched | NA |
| Xbox Kinect (Kinect Adventures and Kinect Sports) and Rutgers V-step | Microsoft | Cerebral palsy and brain injury | Launched | NA |
| Xbox-Kinect, Virtual Reality + treadmill training | Microsoft | Parkinson's disease | Launched | NA |
| YouGrabber | YouRehab Inc. | Stroke | Approved | Europe |
| Wearables | | | | |
| 40 Hz Light therapy system (LTS) | Optoceutics | Alzheimer disease | In development | NA |
| ArmeoSenso - Reward, Game with visual effects and monetary rewards | Hocoma | Stroke rehabilitation | Approved | EU, USA |
| Doppel device | Team Turquoise Ltd | Anxiety | Launched | NA |
| DREEM 2 Headband | DREEM | Alzheimer's disease, dementia | Launched | NA |
| Freespira Breathing System | Palo Alto Health Sciences, Inc | Anxiety and panic disorder | Approved | USA |
| Gait Training (GT) with rhythmic Auditory Stimulation (RAS) | Sponsor: The Cleveland Clinic  Collaborators: Consortium of Multiple Sclerosis Centers, MedRhythms, Inc. | Multiple sclerosis | No information available | NA |
| GENUS (gamma entrainment using sensory stimuli) or synchronized 40 Hz gamma oscillation | Cognito Therapeutics, Inc. | Alzheimer disease | Approved | USA |
| Gloreha Aria | Gloreha IDROGENET | Stroke | Approved | International |
| Hummingbird hand training device | NR | Stroke and cervical spinal cord injury | NA | NA |
| JOGO | JOGOHEALTH | Chronic low-back pain | Approved | USA |
| MR-010 | MedRhythms, Inc | Stroke | In development | NA |
| NerivioMigra app (REN device) | Theranica Bio-Electronics | Acute migraine | Approved | USA |
| NightWare | NightWare | PTSD, co-morbid nightmare disorder | Approved | USA |
| Rapael glove | Neofect | Stroke | Approved | USA, South Korea |
| Rapael kids | Neofect | Brain injury | Approved | USA, South Korea |
| Relivion | Neurolief | Migraine | Approved | USA |
| RGS based training and monitoring | The SPECS research group of IBEC and Eodyne Systems | Stroke | No information available | NA |
| Superpower Glass | Stanford University & Cognoa | ASD | In development | NA |
| The Virtual Reality Rehabilitation System (VRRS) | Khymeia Group | Stroke | Approved | International |
| Computer-based | | | | |
| ACTIVATE | C8 Sciences | ADHD | Launched (temporary access by FDA in Covid 19) | NA |
| Adventures aboard the S.S. GRIN | 3C Institute | Autism (children with social skills challenges) | Launched | NA |
| Aphasia rehabilitation using software program | NR | Aphasia | NA | NA |
| ASCEND-I | NR | Cognitive Impairment/dysfunction | NA | NA |
| Brain HQ | Posit Science | Cognitive Impairment, brain injury, multiple sclerosis, Alzheimer’s disease, schizophrenia | Launched | NA |
| Brain+ Evolution | NR | Parkinson's disease | NA | NA |
| BrainGame Brian | Stichting Gaming & Training | ASD, ADHD | Launched | NA |
| CAVINS (Cognitive Assessment & Video-game Intervention Solutions) | NR | ASD | NA | NA |
| Cogmed Working Memory Training Program | Cogmed | ADHD, cerebral palsy, executive function | Launched | NA |
| CogniPlus and Cogniplus TR | SCHUHFRIED | Stroke, brain injury | Launched | NA |
| Cognitive Stimulation Therapy (CST) program | Brain+ | Dementia | In development | NA |
| CogPack | Marker software | Substance use disorder (alcohol), schizophrenia, psychosis, PTSD | Launched | NA |
| Computer-based training program (CBT for CBT) | CBT4CBT, LLC | Substance use disorder (cocaine) | Launched | NA |
| Computerized cognitive training with customizable avatar | NR | Anxiety | NA | NA |
| Computerized Plasticity-based software | Posit Science | Alzheimer's disease | In development | NA |
| Dojo | GameDesk | Anxiety | No information available | NA |
| Dr Kawashima’s Brain Training | Nintendo | Multiple sclerosis | Launched | NA |
| Emotional Faces Memory Task training (EFMT) | Mount Sinai and Click Therapeutics | Depression | In development | NA |
| FaceSay | Symbionica LLC | ASD | Launched | NA |
| Guardian angel | RETRO Lab at the University of Central Florida | Substance use disorder (alcohol) | NA | NA |
| Lets Face It | University of Victoria, Cognition Lab & Yale Child Study Centre | ASD | NA | NA |
| Lookware | BioStream Technologies | ASD | No information available | NA |
| Mega Team | Sticky brain studios | ADHD, executive function | In development | NA |
| Mindful Garden | MindfulGarden Digital Health, Inc | Delirium | In development | NA |
| Mindlight | GainPlay Studio | Anxiety (children with an ASD) | Launched | NA |
| Odd Yellow WM training | NR | Intellectual disability | NA | NA |
| RehaCom | HASOMED GmbH | Multiple sclerosis, schizophrenia, Stroke | Approved | UK |
| Scientific Brain Training (SBT) | NR | ADHD | NA | NA |
| SPARX | University of Auckland in conjunction with game developers Metia Interactive | Depression | Launched | NA |
| SPARX-R | University of Auckland in conjunction with game developers Metia Interactive | Depression | No information available | NA |
| The Journey | Thatgamecompany and Santa Monica Studio, Sony Interactive Entertainment LLC. | Depression | Launched | NA |
| The Journey to Wild Divine | Wild Divine (now Unyte) | Pediatric anxiety and depression | Launched | NA |
| Visuospatial WM training | NR | ADHD | NA | NA |

ADHD: Attention deficit hyperactivity disorder; ASD: Autism spectrum disorder; CBT: Cognitive behavioral therapy; DTx: Digital therapeutic(s); MDD: Major depressive order; NA: Not applicable; NR: Not reported; PTSD: Post-traumatic stress disorder; QOL: Quality of life; TEC: Therapeutic evaluative conditioning.

*Digital therapeutics listed as medical devices are considered as approved.

Supplementary Table S6: Nature of digital sham

| Study names | Name of digital sham | Name of DTx | Indication | Components of DTx | Components of digital sham | Difference between components of digital sham and DTx | Nature of digital sham based on difference in active component |
| --- | --- | --- | --- | --- | --- | --- | --- |
| Mobile device-based | | | | | | | |
| Berman 2020 | A web-based control app (offering brief information and advice regarding problematic alcohol use) | Telecoach | Alcohol Use Disorder | • A web-based skills training smartphone app  • Three components and their subcomponents deliver coping strategies for excessive drinking –  1. Self-monitoring of alcohol intake and hazardous drinking  2. Saying no to alcohol: Risk situations are identified based on the user’s answers to twelve questions and different ways to say no to alcohol are offered  3. Feeling better without alcohol: Relaxation exercise and positive thoughts | • A web-based smartphone app  • A web-app with information about the health-related consequences of alcohol consumption. | • Unlike the DTx, digital sham **does not offer self-monitoring** • The digital sham only provides information on risky drinking whereas the DTx provides a relaxation exercise, positive thoughts and skills to monitor risky levels of drinking and ways to say no to alcohol | Active component replaced with inactive/neutral component in digital sham |
| Bove 2021 (DigCog) | AKL-T09 | AKL-T03 | Multiple Sclerosis | • A tablet-based videogame  • AKL-T03 is designed using a Selective Stimulus Management Engine (SSME^TM^) which involves the patient in two simultaneous sensory and motor tasks which engage frontal neural networks.  • Fully automatic adjustment of level (dose); personalized and adapted to individual patient needs | • A tablet-based videogame • AKL-T09 is a game in which the user connects letters on a grid to spell as many words as possible. Points are earned by tracing words with two or more letters, in any direction, based on the number of words formed, word length, and use of uncommon letters, with progressive letter grid difficulty. The active placebo control provided similar engagement and time on task | • No difference in components except type of games and active components  • Unlike AKL-T03, AKL-T09 content does **not automatically adjust level (dose)** | Active component in DTx replaced with another active component in digital sham |
| Kollins 2020 (STARS-ADHD) | AKL-T09 | AKL-T01 (EndeavorRx) | ADHD | • A tablet-based (iPad mini 2 tablet) videogame  • The EndeavorRx app requires the subject to navigate a character through a game-like space while collecting objects in a fixed time.  • Difficulty level increases as the game progresses (provides an adaptive and personalized high degree of difficulty)  • Progress is signaled through rewards and unlocking new environments | • A tablet-based (iPad mini 2 tablet) videogame  • AKL-T09 is a game in which the user connects letters on a grid to spell as many words as possible. Points are earned by tracing words with two or more letters, in any direction, based on the number of words formed, word length, and use of uncommon letters, with progressive letter grid difficulty.  • Difficulty increases to maintain engagement and expectation of benefit from patients and their caregivers. However, it does not provide an adaptive and personalized high degree of difficulty. | • No difference in components except **type of games and active components**  • There is progression in difficulty to maintain engagement and expectation of benefit from patients and their caregivers. However, it does **not automatically adjust level (dose) like AKL-T01** | Active component replaced with another active component in digital sham |
| Keefe 2019 (STARS-MDD) | AKL-T09 | AKL-T03 | Major depressive disorder | • A tablet-based videogame • AKL-T03 is designed using a Selective Stimulus Management Engine (SSME^TM^) that involves the patient in two simultaneous sensory and motor tasks which engage frontal neural networks. | • A tablet-based videogame  • AKL-T09 is a is a game in which the user connects letters on a grid to spell as many words as possible. Points are earned by tracing words with two or more letters, in any direction, based on the number of words formed, word length, and use of uncommon letters, with progressive letter grid difficulty. The active placebo control provided similar time on task and engagement. | • No difference in components except type of games and active components  • Unlike AKL-T03, AKL-T09 does **not automatically adjust level (dose)** | Active component replaced with another active component in digital sham |
| Teng 2019 | Placebo training | Home-delivered attentional bias modification (HD-ABM) | Generalized anxiety disorder | • Smartphones (Android phones)  • Threat–neutral word pairs (in Chinese) with a randomly assigned location as left or right visual field of mobile screen with a fixation cross of 5 cm  • ABM app – For the HD-ABM group, the probe was set to replace the neutral stimulus word | • Smartphones (Android phones)  • Threat–neutral word pairs (in Chinese) with a randomly assigned location as left or right visual field of mobile screen with a fixation cross of 5 cm  • ABM app – For the placebo group, the probe was set to replace the neutral or threatening word randomly | For the HD-ABM group, the probe was set to **replace the neutral stimulus word whereas for placebo group, the probe was set to replace the neutral or threatening word randomly** | Active component replaced with inactive/neutral component in digital sham |
| Yerys 2019 | AKL-T09 | Project EVO | Autism Spectrum Disorder and ADHD | • A tablet-based videogame  • Project EVO app installed on iPad | • A tablet-based videogame  • AKL-T09, Project EVO’s non-multi-tasking, educational-based intervention (“educational”) requires children to generate words from an array of letters. Children were rewarded for generating longer words, and so task difficulty was self-imposed by children’s ability and desire to generate longer words. | Unlike Project EVO, the sham control is **non-multi-tasking and non-adaptive/ personalized for a high degree of difficulty** | Active component in DTx replaced with another active component in digital sham |
| Bucci 2018 | ClinTouch app | Actissist | Psychosis | • Smartphone app  • Delivers CBT for early psychosis  • Actissist consists of 5 intervention domains (voices, suspicious thoughts, socializing, criticism, and cannabis)  • Multimedia intervention components include symptom fact sheets, a diary, guided relaxation and mindfulness exercises, patient recovery video stories, links to relevant resources, and emergency contacts.  • Actissist consists of tailored normalizing messages, and cognitive or behavioural coping strategies.  • Actissist also features a user-customizable display (users set app wallpaper from the smartphone photo album) | • Smartphone app  • ClinTouch app only monitors psychosis and mood symptoms without providing self-managed therapy.  • As in the treatment condition, the app emits an alarm prompting participants to access the app at 3 pseudo-randomized time points per day, 6 days a week. | DTx provides tailored normalizing messages and cognitive or behavioural coping strategies whereas the digital sham **only monitors psychosis and mood symptoms but does not provide self-managed therapy.** | No active component in digital sham |
| Deady 2018 | HeadGear lite app | HeadGear | Depression | • Apple/Android-operating smartphone  • Internet  • The Headgear app includes ‘challenges’ which incorporate a variety of evidence-based BAT and mindfulness techniques and skills, including psychoeducational videos, value-driven activity planning and goal-setting, practice exercises, and techniques for developing coping and resilience.  • Mood monitoring, a skill ‘toolbox’ (progressively built as the skills are completed), and a technical service helpline. | • Apple/Android-operating smartphone  • Internet  • HeadGear lite app (attention-matched control) has an inbuilt mood monitor daily and access to the ‘risk calculator but NO skill development and no component of behavioural activation or mindfulness therapy  • Also includes the risk calculator, daily mood monitoring and personalized feedback regarding risk for future mental health issues. | The attention-matched control condition is a smartphone application that will have the same name and a virtually identical look and ‘feel’ as the intervention version of Headgear, accessed in the same manner. However, there is **no skill development and no component of behavioural activation or mindfulness therapy.** | No active component in digital sham |
| Davies 2017 | Imagination of information about alcohol misuse | Drinks Meter | Risky drinking | • Smartphone app  • Online digital app that offers a traditional ‘identification and brief advice’ approach in which users receive personalized feedback about their own drinking and are told how it compares with other people with similar demographic/geographic characteristics.  • Provides information about the number of calories they consume when drinking and the amount of money they spend on alcohol | • Imaginary condition  • Participants were directed to a placebo condition, where they were asked to imagine they are exposed to (reading/watching/ listening to) information about alcohol misuse, without actually receiving any alcohol information.  • This was intended to act as a more robust placebo condition, as the active ingredients of the intervention will have been removed (e.g. feedback on drinking), but other aspects e.g. being directed to a website and being given some information about alcohol, remained (albeit in an imaginary sense). | Drinks Meter provides online app-based actual information on alcohol use, drink monitoring, brief advice, and personalized feedback on consumed calories and the amount of money spent on alcohol; whereas the placebo consists of **imaginary information about alcohol misuse, without any real information**. | Active component replaced with inactive/neutral component in digital sham |
| Dennis-Tiwary 2017 | Placebo – attention training application | Personal Zen (ABMT) | Stress and anxiety during pregnancy | • Smartphone  • ABMT application  • ABMT protocol (dot probe) incorporating appealing video game-like features such as animated characters and sound effects  • Like traditional ABMT, attention is systematically redirected away from threat-relevant stimuli (angry faces), but in a more appealing and engaging format.  • Stimuli for the dot probe task are pictures of 20 different individuals (10 males, 10 females). Stimuli were programmed using E-Prime version 2.0 | • Smartphone  • Placebo training application  • ABMT protocol (dot probe) incorporating appealing video game-like features such as animated characters and sound effects | In the ABMT version, a trail of grass appeared in the location of the non-threat character for every trial, whereas in the placebo training version, **a trail was equally likely to appear in the location of the angry or neutral sprite.** | Active component replaced with inactive/ neutral component in digital sham |
| Giosan 2017 | Placebo app against Dcombat | Dcombat | Depression | • Smartphone app (iOS and Android)  • Dcombat is an innovative smartphone app that uses the basic principles of CBT in treating depressive symptoms. The mHealth CBT intervention comprises of four online intervention modules with specific courses and exercises, activated gradually, a specific assignment for each module, regular automatic messages and assessments, and a therapist check-in for each module. | • Smartphone app (iOS and Android)  • The active placebo intervention is delivered via the same platform and largely in the same format as the tested app: it will include largely the same sections and features as the original app (i.e., profile, courses, exercises, evaluation, and messages) but will include different content without the active therapeutic component | Dcombat includes psychoeducational materials and exercises based on CBT for depression, but the placebo includes **different content (elaborating on common-sense strategies of overcoming depression like positive thinking, listening to music, etc. and general knowledge about mental and physical health topics)** | Active component replaced with inactive/ neutral component in digital sham |
| Franklin 2016 | TEC with neutral (or blank) images | TEC | Self-injurious thoughts and behaviors | • Smartphone (can also be assessed via phones, tablets, laptops, and desktops)  • Game-based  • Several TEC characteristics promoted this aim: It takes 1 to 2 min to complete a single instance of TEC; TEC becomes more challenging as the trials progress; points are awarded for faster and more accurate performance; each instance of TEC is unique, increasing replay value; and although TEC was primarily intended as a mobile app. | • Smartphone (can also be assessed via phones, tablets, laptops, and desktops)  • Game based  • This TEC version included almost same features but replaced active (positive) images with neutral (or blank) images. | Same components are there in both however in sham TEC version **active (positive) images are replaced with neutral (or blank) images**. | Active component of DTx replaced with non-active component in digital sham |
| Enock 2014 | Control training (no contingency training) | Attention Bias Modification Training | Anxiety (social anxiety) | • Smartphone (iPhone, iPod Touch, or Android-based phone with Wi-Fi or other Internet access)  • Dot-probe task – involving faces with ‘neutral’ and ‘disgust’ (representative of social threat) expressions – included a contingency to induce attentional deployment away from ‘disgust’ faces. | • Smartphone (iPhone, iPod Touch, or Android-based phone with Wi-Fi or other Internet access)  • Dot-probe task – involving ‘neutral’ and ‘disgust’ faces; ) – the control training included no contingency | Unlike DTx, digital sham **does not include a contingency to induce attentional deployment away from ‘disgust’ faces.** | Active component replaced with inactive/ neutral component in digital sham |
| NCT03751280 | PEAR-004 placebo app | PEAR-004 | Schizophrenia | • Smartphone app (iOS and Android based)  • Pear-004 is a patient-facing application and clinician-facing web interface  • Pear-004, supports in setting, achieving weekly goals and learning skills to help manage symptoms and challenges associated with psychotic disorders (schizophrenia, schizoaffective disorder, bipolar I/II with psychotic features and depression with psychotic features)  • The participants assigned the PEAR-004 app downloaded an app called “Thrive” on their smartphone  • The app notified the participants when to open it each day. When opened, it delivered information to help build skills for coping with unwanted thoughts or voices, depression, and problems with social relationships | • Smartphone app (iOS and Android based)  • PEAR-004 placebo app looked like the PEAR-004 app but did not have any therapy content  • The placebo app notified participants when to open it each day. The app had a timer that showed how much time was left until the end of trial treatment. When the participants opened the app, they would only see the timer and no therapy content. | PEAR-004 placebo app looked like the PEAR-004 app but **did not have any therapy content** | No active component in digital sham |
| NCT02828644 | AKL-T09 | AKL-T01 (EndeavorRx) | ADHD | • A tablet-based (iPad mini 2 tablet) videogame  • The EndeavorRx app requires the subject to navigate a character through a game-like space, while collecting objects, in a fixed time.  • Difficulty level increases as the game progresses (provides an adaptive and personalized high degree of difficulty)  • Progress is signaled through rewards and unlocking new environments | • A tablet-based (iPad mini 2 tablet) videogame  • AKL-T09 is a game in which the user connects letters on a on a grid to spell as many words as possible. Points are earned by tracing words with two or more letters, in any direction, based on the number of words formed, word length, and use of uncommon letters, with progressive letter grid difficulty.  •Difficulty increases to maintain engagement | • No difference in components except nature of games and active components  • There is progression in difficulty to maintain engagement and expectation of benefit from patients and their caregivers. However, it **does not automatically adjust level (dose) like AKL-T01** | Active component replaced with inactive/neutral component in digital sham |
| NCT04779372 | Online sleep education/ Sleep Hygiene Education | WeChat app – eCBTI | Insomnia | • This is a smartphone-based dCBT-I from a WeChat applet | Patients receive sleep education advice from common sleep clinic by the same applet as the group of CBT-I in smartphone | Patients receive sleep education **advice from common sleep clinic by the same applet as the group of CBT-I in smartphone** | Active component replaced with inactive/neutral component in digital sham |
| Web-based | | | | | | | |
| Cheng 2021 (SPREAD trial) | Online sleep education/ Sleep Hygiene Education | Sleepio | Insomnia | • Web-based  • Fully online  • Delivered by an animated “virtual therapist” (The Prof).  • No face-to-face contact  • Sleep education and hygiene, relaxation, Behavioural techniques (e.g., sleep restriction, stimulus control), cognitive techniques (restructuring, paradox, mindfulness, imagery, putting day to rest, thought stopping)  • Appointment system, interactive sessions, dynamic feedback against personal goals, progress review at start of each session, automatic calculation of sleep data over time, personal case file, end of session quiz, 24/7 access  • Support/ motivational system: Praise/reinforcement contingent on progress, online Wikipedia of sleep educational topics, social community of users, moderated by experts, support/prompts/reminders by email and mobile SMS, ‘graduation ceremony’ on course completion | • A web-based education program delivered by email or on a dedicated website where materials can be viewed and downloaded | In the Sleepio group, the intervention is delivered by animated prof whereas in sham control, the intervention is delivered by email or on a dedicated website where materials can be viewed and downloaded, **not in a personally tailored manner**.  • Furthermore, the sham control **did not deliver the active component which included cognitive and behavioural techniques to improve sleep quality** | Active component replaced with inactive/neutral component in digital sham |
| Kalmbach 2020 | Online sleep education/ Sleep Hygiene Education | Sleepio | Insomnia in pregnancy | • Web-based  • Fully online  • Delivered by an animated “virtual therapist” (The Prof).  • No face-to-face contact  • Sleep education and hygiene, relaxation, Behavioural techniques (e.g., sleep restriction, stimulus control), cognitive techniques (restructuring, paradox, mindfulness, imagery, putting day to rest, thought stopping)  •Appointment system, interactive sessions, dynamic feedback against personal goals, progress review at start of each session, automatic calculation of sleep data over time, personal case file, end of session quiz, 24/7 access  •Praise/reinforcement contingent on progress, online Wikipedia of sleep educational topics, social community of users, moderated by experts, support/prompts/reminders by email and mobile SMS, ‘Graduation ceremony’ on course completion | • A web-based education program delivered by email  • Sleep information (derived from the Sleepio library)  • Delivered by email or on a dedicated website where materials can be viewed and downloaded | • In the Sleepio group, the intervention is delivered by animated prof whereas in sham control, the intervention is delivered by email or on a dedicated website where materials can be viewed and downloaded, **not in a personally tailored manner**.  • Furthermore, the sham control **did not deliver the active component which included cognitive and behavioural techniques to improve sleep quality** | Active component replaced with inactive/neutral component in digital sham |
| Espie 2019 (DIALS study) | Online sleep education/ Sleep Hygiene Education | Sleepio | Insomnia | • Web-based  • Fully online  • Delivered by an animated “virtual therapist” (The Prof).  • No face-to-face contact  • Sleep education and hygiene, relaxation, Behavioural techniques (e.g., sleep restriction, stimulus control), cognitive techniques (restructuring, paradox, mindfulness, imagery, putting day to rest, thought stopping)  •Appointment system, interactive sessions, dynamic feedback against personal goals, progress review at start of each session, automatic calculation of sleep data over time, personal case file, end of session quiz, 24/7 access  • Praise/reinforcement contingent on progress, online Wikipedia of sleep educational topics, social community of users, moderated by experts, support/prompts/reminders by email and mobile SMS, ‘graduation ceremony’ on course completion | • A website and a downloadable booklet  • Sleep information (derived from the Sleepio library)  • Delivered by email or on a dedicated website | • In the Sleepio group, the intervention is delivered by animated prof whereas in sham control, the intervention is delivered by email or on a dedicated website where materials can be viewed and downloaded, **not in a personally tailored manner**.  • Furthermore, the sham control **did not deliver the active component which included cognitive and behavioural techniques to improve sleep quality** | Active component replaced with inactive/neutral component in digital sham |
| Batterham 2018 | HealthWatch | FitMindKit | Mood, anxiety, substance use and suicidality | • A web-based program  • Consists of ten core modules based narrative approach to introduce the core strategies of relevant behavioural therapies, modules tailored to their specific mental health symptoms | • A web-based program  • Text based-modules containing information on bone health, sun exposure, food hygiene, use of vitamins and supplements, kidney health, microbes, household burns, respiratory viruses, heart health, and allergens. | • HealthWatch modules were **fully text-based** whereas FitMindKit modules also included brief (2–6 min) videos.  • Unlike FitMindKit, HealthWatch provides modules in a **non-tailored condition and does not target the behavioural therapies** | Active component replaced with inactive/neutral component in digital sham |
| Parks 2018 | Psychoeducation | Happify | Anxiety and depression | • Web-based (smartphone, laptop, desktop)  • Offers techniques grounded in positive psychological interventions (PPI), CBT, and/or mindfulness-based stress reduction  • Fully online | • Web-based (smartphone, laptop, desktop)  • Psychoeducational participants logged in regularly to an identical looking Happify website or app, included an identical onboarding process, and were offered content that grew and changed over time  • Fully online | Psychoeducation group users did not experience some of the engagement elements from the main platform—they had no access to social forums, were unable to post their activity results publicly for others to see and comment on, or to choose freely between different programs | Active component replaced with inactive/neutral component in digital sham |
| Denis 2017 | Puzzles | Sleepio | Insomnia | • A web-based program  • Fully online  • Delivered by an animated “virtual therapist” (The Prof)  • No face-to-face contact  • Sleep education and hygiene, relaxation, behavioural and cognitive techniques  • Appointment system, interactive sessions, dynamic feedback against personal goals, progress review at start of each session, automatic calculation of sleep data over time, personal case file, end of session quiz, 24/7 access  • Praise/reinforcement contingent on progress, online Wikipedia of sleep educational topics, social community of users, moderated by experts, support/prompts/reminders by email and mobile SMS, ‘graduation ceremony’ on course completion | • A web-based program  • Puzzles were directly sent to participants via automated distribution emails, at 7-day intervals  • The types of puzzles included word searches, crosswords, and lateral thinking problems | • Unlike digital sham, DTx program covers behavioural (e.g., sleep restriction, stimulus control) and cognitive (e.g., putting the day to rest, thought restructuring, imagery, articulatory suppression, paradoxical intention, mindfulness) strategies  • Unlike digital sham, DTx program comprised a **fully automated media-rich web application and an animated “virtual therapist” (The Prof)**  • Unlike digital sham,  DTx program also includes **sleep diary as a part of the intervention filled by participants on daily basis** | Active component replaced with inactive/neutral component in digital sham |
| Perry 2017 (TriPoD) | lifeSTYLE | SPARX-R | Depression | • Web based (desktop computers via the internet)  • Supplemented with a paper notebook for students to review key messages from each module and record personal comments  • An interactive, avatar-based fantasy game delivering CBT, designed to prevent, and treat depression in young people. | • Web based (desktop computers via the internet)  • lifeSTYLE is an adaptation of an interactive, online program which includes seven modules (approximately 25 minutes each) and covers the following topics: (1) independence, (2) participating in your community, (3) work skills, (4) mobile phone safety and hygiene, (5) healthy skin, (6) sustainable eating and (7) maintaining a healthy home environment. Each module includes information about the specified topic as well as interactive activities such as quizzes, myth busters, videos, and scenarios that students can reflect on and respond to. | SPARX-R is an avatar-based fantasy game format that provides CBT, but sham (lifeSTYLE) control provided **only educational material and online open-ended questions that probe health factors,** physical and artistic activities, education and hobbies, social, financial, and family roles, work habits and stress, medications, pain, and nutrition. | Active component replaced with another active component in digital sham |
| Zwerenz 2017 | Online information about depression | Deprexis | Depression | • Web-based intervention  • Delivered online (can be assessed using laptop, desktop, or smartphone)  • Deprexis includes10 main modules plus 1 introductory and 1 summary module. All modules include drawings, photographs and audio-recorded guided imagery and relaxation exercises.  • The eclectic program uses techniques like cognitive behavioural psychotherapy, positive psychology, emotion-focused therapy, and dream work.  • Optional reminders via email and SMS can be activated. | • Web-based intervention  • Delivered online (can be assessed using laptop, desktop, or smartphone)  • An online platform providing 12 weekly modules with specific topics regarding depression  • To ensure comparability with the intervention group, the sham group was also given 2 different weekly time slots of equal length in their treatment plan to use the online platform. | Deprexis program uses different psychotherapeutic orientations like cognitive behavioural psychotherapy, positive psychology, emotion-focused therapy, and dream work whereas sham control **just delivers online information about depression** | Active component replaced with inactive/neutral component in digital sham |
| Christensen 2016 (GoodNight Study) | HealthWatch | SHUTi | Insomnia and depression | • A web-based program  • SHUTi provides automated cognitive behavioural therapy for insomnia that is tailored and interactive.  • Six sequential modules about insomnia, and two behavioural modules about sleep restriction and stimulus control, cognitive restructuring, sleep hygiene, and relapse prevention. | • A web-based program  • The HealthWatch program has no specific mental health or sleep-related content.  •HealthWatch modules includes information on environmental health, nutrition myths, heart health, activity, medication, etc. | Unlike SHUTi (6-week sequential module), Healthwatch is available in various versions (i.e. 9, 10 and 12 weeks etc.) and **do not deliver CBT for insomnia** | Active component replaced with inactive/neutral component in digital sham |
| Deady 2016 | HealthWatch | DEAL Project (Depression Alcohol) intervention | Depression and Problematic Alcohol Use | • A web-based program  • Computer (or other preferred port for Internet access)  • The DEAL Project based on the SHADE program (Self-Help for Alcohol and other drugs and Depression), consists of evidence-based cognitive behavioural therapy and motivational interviewing.  • Four 1-hour modules to be completed over a 4-week period  • Automated email reminders sent to participants | • A web-based program  • Computer (or other preferred port for Internet access)  • The HealthWatch program has no specific mental health or sleep-related content.  • 12-module | HealthWatch consists of more modules and **does not deliver any cognitive behavioural therapy and motivational interviewing** | Active component replaced with inactive/neutral component in digital sham |
| Pinheiro 2016 | Online sleep education/ Sleep Hygiene Education | Sleepio | Insomnia in patients with lower back pain | • Web-based  • Fully online  • Delivered by an animated “virtual therapist” (The Prof).  • No face-to-face contact  • Sleep information/education, sleep hygiene, relaxation, Behavioural techniques (e.g., sleep restriction, stimulus control), cognitive techniques (restructuring, paradox, mindfulness, imagery, putting day to rest, thought stopping)  • Appointment system, interactive sessions, dynamic feedback against personal goals, progress review at start of each session, automatic calculation of sleep data over time, personal case file, end of session quiz, 24/7 access  • Praise/reinforcement contingent on progress, online Wikipedia of sleep educational topics, social community of users, moderated by experts, support/prompts/reminders by email and mobile SMS, ‘graduation ceremony’ on course completion | • A web-based education program delivered by email  • Sleep information (derived from the Sleepio library)  • Delivered by email or on a dedicated website where materials can be viewed and downloaded | • In the Sleepio group, the intervention is delivered by animated prof whereas in sham control, the intervention is delivered **by email or on a dedicated website** where materials can be viewed and downloaded**, not in a personally tailored manner**.  • Furthermore, the sham control **did not deliver the active component which included cognitive and behavioural techniques to improve sleep quality** | Active component replaced with inactive/neutral component in digital sham |
| Espie 2012 | Imagery relief therapy (IRT) | Sleepio | Insomnia | • Web based  • Fully online  • No face-to-face contact • Delivered by an animated “virtual therapist” (The Prof)  • Sleep information/education, sleep hygiene, relaxation, Behavioural techniques (e.g., sleep restriction, stimulus control), cognitive techniques (restructuring, paradox, mindfulness, imagery, putting day to rest, thought stopping)  • Appointment system, interactive sessions, dynamic feedback against personal goals, progress review at start of each session, automatic calculation of sleep data over time, personal case file, end of session quiz, 24/7 access.  • Praise/reinforcement contingent on progress, online Wikipedia of sleep educational topics, social community of users, moderated by experts, support/prompts/reminders by email and mobile SMS, ‘graduation ceremony’ on course completion | • Smartphone app (iOS and Android)  • Fully online  • No face-to-face contact  • Delivered by animated therapist (The Prof)  • Sleep information/education, hierarchy development, imagery training, scheduled pseudo-desensitization, breathing control  • Appointment system, interactive sessions, dynamic feedback against personal goals, progress review at start of each session, automatic calculation of sleep data over time, personal case file, 24/7 access  • Praise/reinforcement contingent on progress, online Wikipedia of sleep educational topics, support/prompts/reminders by email and mobile SMS, ‘graduation ceremony’ on course completion | • Imagery relief therapy was also delivered by The Prof, using the same application platform, and design and execution principles as for CBT, but with **no known active therapeutic ingredient**. | Active component replaced with inactive/neutral component in digital sham |
| NCT03322774 (STRIDE) | Online sleep education/ Sleep Hygiene Education | Sleepio | Insomnia | • Web-based  • Fully online  • Delivered by an animated “virtual therapist” (The Prof).  • No face-to-face contact (the program comprised a fully automated media-rich web application, driven dynamically by baseline, adherence, performance, and progress data)  • Sleep information/education, sleep hygiene, relaxation, Behavioural techniques (e.g., sleep restriction, stimulus control), cognitive techniques (restructuring, paradox, mindfulness, imagery, putting day to rest, thought stopping)  • Appointment system, interactive sessions, dynamic feedback against personal goals, progress review at start of each session, automatic calculation of sleep data over time, personal case file, end of session quiz, 24/7 access  • Praise/reinforcement contingent on progress, online Wikipedia of sleep educational topics, social community of users, moderated by experts, support/prompts/reminders by email and mobile SMS, ‘graduation ceremony’ on course completion | • A web-based education program delivered by email  • Delivered by email or on a dedicated website where materials can be viewed and downloaded  • In Step 1, sleep hygiene control will be exclusively online. In Step 2, sleep hygiene control includes an in-person, face-to-face component. | In the Sleepio group, the intervention is delivered by animated prof whereas in sham control, the intervention is **delivered by email or delivered on a dedicated website** where materials can be viewed and downloaded, **not in a personally tailored manner.**  • Furthermore, the sham control **did not deliver the active component which included cognitive and behavioural techniques to improve sleep quality** | Active component replaced with inactive/neutral component in digital sham |
| NCT03613519 | Online sleep education/ Sleep Hygiene Education | SHUTi | Insomnia | • A web-based program • SHUTi provides six sequential modules consisting of an overview of insomnia, and two behavioural modules focusing on sleep restriction and stimulus control, cognitive restructuring, sleep hygiene, and relapse prevention. | • A web-based program  • Delivered by email or delivered on a dedicated website where materials can be viewed and downloaded, not in a personally tailored manner  • Sleep information (derived from the Sleepio library), Online sleep education on the following topics: the basics of endogenous sleep regulation; the impact on sleep of health problems; the effects of sleep disruptive substances, such as caffeine, nicotine, and alcohol; and tips on creating a sleep-conducive bedroom environment. | • Unlike SHUTi, digital sham is **not available in a personally tailored manner**  • Furthermore, the sham control **did not deliver the active component which included cognitive and behavioural techniques to improve sleep quality** | Active component replaced with inactive/neutral component in digital sham |
| NCT03724305 (RESIST) | Online sleep education/ Sleep Hygiene Education | Sleepio | Insomnia | • A web-based program  • Fully online  • Delivered by an animated “virtual therapist” (The Prof).  • No face-to-face contact  • Sleep information/education, sleep hygiene, relaxation, behavioral techniques (e.g., sleep restriction, stimulus control), cognitive techniques (restructuring, paradox, mindfulness, imagery, putting day to rest, thought stopping)  • Appointment system, interactive sessions, dynamic feedback against personal goals, progress review at start of each session, automatic calculation of sleep data over time, personal case file, end of session quiz, 24/7 access  • Praise/reinforcement contingent on progress, online Wikipedia of sleep educational topics social community of users, moderated by experts, support/prompts/reminders by email and mobile SMS, ‘graduation ceremony’ on course completion | • A web-based education program delivered by email  • Sleep information  • Delivered by email or delivered on a dedicated website where materials can be viewed and downloaded, not in a personally tailored manner | • In the Sleepio group, the intervention is delivered by animated prof whereas sham control intervention, the is **delivered by email** or delivered on a dedicated website where materials can be viewed and downloaded**, not in a personally tailored manner.**  • Furthermore, the sham control **did not deliver the active component which included cognitive and behavioural techniques to improve sleep quality** | Active component replaced with inactive/neutral component in digital sham |
| NCT04112225 | Psychoeducation | Happify | Loneliness | • Web-based (Smartphone, laptop, desktop)  • Fully online | • Web-based (Smartphone, laptop, desktop)  • Fully online | The psychoeducation control group participants used a version of the Happify platform that encourages thinking about well-being through quizzes and polls without providing any instructions for promotion (i.e., wait-list control). | Active component replaced with inactive/neutral component in digital sham |
| Extended reality | | | | | | | |
| Bentz 2021 | Google Street View app | Easy Heights | Fear of heights | • Smartphone  • Easy Heights app (XR exposure app)  • Noise cancelling headphones  • A Google Daydream View version 2 VR headset  • Provides 16 height stimuli 16 levels based on three different scenarios (rural mountain, cloudy weather, urban town) ranging from 0 and 75 m  • Delivers avoidance behavior and subjective fear in a real-life height situation | • Smartphone  • Google Street View app that explores three predefined virtual scenarios (Iglou visitor center, Versailles, cubic houses) in XR  • Noise cancelling headphones  • A Google Daydream View version 2 VR headset | Unlike DTx, digital sham **did not consist of virtual scenarios targeting any height stimuli** | Active component replaced with inactive/neutral component in digital sham |
| Garcia 2021 | EaseVRx headset without active intervention | EaseVRx headset with active intervention | Chronic Lower back Pain | • Pico G2 4K headset  • XR software developed by AppliedVR on the basis of cognitive behavioural therapy principles, biofeedback, and mindfulness strategies for pain management.  • A 3D, skills-based, interactive software with microphone embedded in the Pico G2 hardware. | • Pico G2 4K headset  • XR software developed by AppliedVR with neutral non-interactive2D nature videos, displayed in a void theatre | • Unlike DTx, digital sham is a **non-interactive, and non-immersive XR therapy.** Instead of 360-degree, 3D, interactive content in DTx, **digital sham consists of only 2D nature footage with neutral music layered on top that is selected to be neither overly relaxing nor distracting** | Active component replaced with inactive/neutral component in digital sham |
| Smeijers 2019 | VR placebo game | Virtual Reality Game for Aggression Impulsive Management (VR-GAIME) | Aggression regulation problems | • XR computer game  • The participants are trained to respond with avoidance behavior to anger-relevant situations.  The participant is met by avatars who are acting in either an agreeable or disagreeable manner, patients will be instructed to lean forward (approach) in response to agreeable avatars and to lean backwards (i.e., avoidance) in response to disagreeable avatars. | • XR computer game  • Game – Patients will play the same game as patients in the experimental condition. However, patients in the control group will not encounter any disagreeable avatars and will hence not receive any training about anger-relevant situations. | Unlike the DTx, sham control group participants, **do not encounter any disagreeable avatars and hence do not receive any training about anger-relevant situations.** | Active component replaced with inactive/neutral component in digital sham |
| McEwen 2014 | Control, virtual reality system (IREX) | Virtual Reality system (IREX) | Stroke | • XR Interactive Rehabilitation Exercise software  • XR games (e.g., soccer goaltending, snowboarding) in a standing position | • XR Interactive Rehabilitation Exercise software  • Patients were seated and played games that did not require any weight shifting within their base of support | Unlike DT, digital sham consists of games which **did not challenge balance (performed in sitting)** | Active component replaced with inactive/neutral component in digital sham |
| In 2012 | Control (sham program) | Virtual reality reflection therapy program | Chronic stroke | • Personal computer  • Camera  • XR reflection equipment  • Each patient in the experimental group had their affected hand put in the box while the other one was placed directly under the camera | • Personal computer  • Camera  • XR reflection equipment  • The control group received the same treatment as active group, except they had to look at the unaffected hand as the monitor was off. | The control group received the same treatment as active group, **except they had to look at the unaffected hand as the monitor was off.** | Active component replaced with inactive/neutral component in digital sham |
| NCT04714645 | Sham XR training | Oculus Rift (HMD VR + Training) | Stroke | • Head-mounted display XR  • A XR training program on unilateral spatial neglect (USN) after stroke | • Head-mounted display XR  • A virtual environment without a specific task designed for neglect training | Digital sham **does not include virtual environment based specific task designed for neglect training** | Active component of DTx missing in digital sham |
| NCT03135418 | XR program only focusing on visual scenes without upper extremity interaction | Oculus Rift and Leap motion | Stroke | • 3D XR headsets  • Oculus Rift and Leap motion based immersive interactive environment  • Task-oriented games like bowling, arrow shooting and home activity simulations which focuses upper extremity abduction, flexion, and rotation movements. | • 3D XR headsets  • Oculus Rift based immersive visual environment  • The XR program only focusing on visual scenes without upper extremity interaction like bobath therapy, walking exercises, upper extremity active exercises, proprioceptive neuromuscular facial techniques. | • Unlike DTx, control group receive a sham XR on **focusing visual scenes without upper extremity interaction** | Active component replaced with inactive/neutral component in digital sham |
| NCT03458611 | Steady central looming stimulus | Virtual Reality Attention Training (VRAT) | Stroke | • XR software  • Provide a multisensory looming stimulus (visual and auditory modality) to orient patients towards the contralesionally side than in the ipsilesional field  • Provides reward for detecting targets on this side | • XR software  • The stimulus presentation is only located in the center of the visual field | The active and placebo intervention were identical in all aspects except for the fact that **sham control consisted of the stimulus presentation in the center of the visual field**, whereas multisensory looming stimuli in **VRAT was more frequently in the contralesionally field** than in the ipsilesional field | Active component replaced with inactive/neutral component in digital sham |
| NCT04225884 | Sham XR software for chronic pain | Virtual Reality Software for Chronic Pain (VIRPI) | Chronic Lower back Pain | • XR software  • With active component for pain (limited data) | • XR software  • Without active component for pain | Unlike DTx, digital sham **does not deliver active intervention for pain** | Active component of DTx missing in digital sham |
| NCT04443127 | Motor-Relearning Program | Virtual Reality Training Session- Game-Based Rehabilitation | Stroke | • XR training session  • XR based motor priming intervention on two XR environments | • XR environments  • Motor-relearning program intervention on two XR environments | DTx is an XR-based **motor priming** intervention while in sham control is a **motor-relearning program intervention** | Active component replaced with inactive/neutral component in digital sham |
| NCT02066116 | Sham virtual rehabilitation education | Kinect-based Rehabilitation | Stroke | • XR therapy  • A Kinect-based upper extremity rehabilitation program | • XR  • A computer-based cognitive rehabilitation program | • Unlike DTx, in digital sham group, patients were encouraged to use their affected arm to push the button, but the **motion remains minimal and not related to the concept of upper extremity rehabilitation**.  • DTx delivers Kinect-based rehabilitation whereas sham group delivers self-exercises education | Active component replaced with inactive/neutral component in digital sham |
| NCT01677429 | Still pictures from XR | Computer Assisted Rehabilitation Environment (CAREN) | Panic disorder | • XR movie + balance challenge | • Still pictures from XR | In DTx, XR movie was shown, while in sham, still pictures were shown. Unlike DTx, **digital sham does not deliver any balance challenge** | Active component of DTx missing in digital sham |
| NCT04139564 | EaseVRx headset without active intervention | EaseVRx headset with active intervention | Chronic Lower back Pain | • XR headset  • XR software comprising seven interactive and 3D modules based on principles of CBT, pain psychology, mindfulness-based stress reduction, biofeedback, and distraction therapy commonly used in interdisciplinary pain rehabilitation programs. | • XR headset  • XR software with neutral non-interactive 2D visual wildlife scenes similar to some EaseVRx content | Unlike DTx, digital sham was a non-interactive XR therapy containing **only 2D** visual wildlife scenes like some EaseVRx content | Active component replaced with inactive/neutral component in digital sham |
| Wearables | | | | | | | |
| Cimenser 2021 | GENUS (gamma entrainment using sensory stimuli): Sham Settings | GENUS (gamma entrainment using sensory stimuli) or synchronized 40 Hz gamma oscillation | Alzheimer's Disease | • Proprietary auditory and visual sensory stimulation device  • Active GammaSense Stimulation System | • Proprietary auditory and visual sensory stimulation device  • Sham GammaSense Stimulation System | The sham condition was similar to active sensory stimulation but did not evoke cortical 40 Hz steady-state oscillations. | Active component replaced with inactive/neutral component in digital sham |
| Pasula 2020 | NerivioMigra app (REN device) - Sham stimulation | NerivioMigra app (REN device) | Acute migraine | • A wireless wearable battery-operated stimulation device  • A smartphone software application controlling stimulation | • A wireless wearable battery-operated device with different stimulating pulse frequency  • A smartphone software application controlling stimulation | The sham device had a pulse frequency and width of ~0.083 Hz and 40-550 μs (modulated), which was thus aimed to induce a solid and perceptible sensation almost like the active device, but with a sufficient low frequency to stop any modulation of nociceptive processes. | Active component replaced with inactive/neutral component in digital sham |
| Schenker 2019 | Relivion Device- Sham stimulation | Relivion | Migraine | • A wearable device (that delivers electric stimulation to nerves) + e-Relief mobile app (that displays and transmits information between patients and the cloud) | • A wearable device (Headset)  • Sham occipital and supraorbital transcutaneous nerve stimulation + e-Relief mobile app | Same components except **lower treatment intensity** in sham control | Low intensity treatment in digital sham |
| Azevedo 2017 | Doppel device with active component (Doppel) turned off condition | Doppel device | Anxiety during the anticipation of public speech | • A wristband  • Delivers heartbeat-like tactile stimulation based calming effect of Doppel on physiological arousal and subjective experience during a socially stressful situation. | • Same Doppel wristband was used with the active component (Doppel) turned off | Sham control consists of same Doppel device with the **active component (Doppel) turned off** | Active component turned off in digital sham |
| NCT03185559 | Relivion Device- Sham stimulation | Relivion | Migraine | • A wearable device (that delivers electric stimulation to nerves) + e-Relief mobile app (that displays and transmits information between patients and the cloud) | • A wearable headset  • Sham occipital and supraorbital transcutaneous nerve stimulation + e-Relief mobile app | Same components except **lower treatment intensity** in sham control | Low intensity treatment in digital sham |
| NCT03631550 (The RIME Study) | Relivion Device- Sham stimulation | Relivion | Migraine | • A wearable device (that delivers electric stimulation to nerves) + e-Relief mobile app (that displays and transmits information between patients and the cloud) | • A wearable headset  • Sham occipital and supraorbital transcutaneous nerve stimulation + e-Relief mobile app | Same components except **lower treatment intensity** in sham control | Low intensity treatment in digital sham |
| NCT03934658 | Sham NightWare | NightWare | Post-Traumatic Stress Disorder, Co-Morbid Nightmare Disorder | • iPhone + Apple watch + proprietary application [senses physiologic parameters, the participant is aroused from sleep (without awakening) so that the nightmare is interrupted prior to reaching a threshold of severity in which the participant would awaken in distress. Seconds later the participant returns to sleep without experiencing a nightmare.] | • Wearable (iPhone + Apple watch + proprietary application)  • No physiologic parameters sensing based automated vibration | Digital sham **does not include physiologic parameters sensing based automated vibration** | Active component turned off in digital sham |
| NCT04040387 | Sham NightWare | NightWare | Post-Traumatic Stress Disorder, Co-Morbid Nightmare Disorder | • iPhone + Apple watch + proprietary application [senses physiologic parameters, the participant is aroused from sleep (without awakening) so that the nightmare is interrupted prior to reaching a threshold of severity in which the participant would awaken in distress. Seconds later the participant returns to sleep without experiencing a nightmare.] | • Wearable (iPhone + Apple watch + proprietary application)  • No physiologic parameters sensing based automated vibration | Digital sham **does not include physiologic parameters sensing based automated vibration** | Active component turned off in digital sham |
| Computer based | | | | | | | |
| Sosnowski 2021 | Control video game | Lookware™ | Autism Spectrum Disorder | • Consumer grade Laptop  • Low-cost Tobii 4C eye tracker  • Xbox game controller  • Gameplay sessions are comprised of a therapeutic exercise phase and a reward phase that cycle back and forth several times during each session | • Laptop  • Eye tracker  • Game controller  • The intervention’s therapeutic exercises are not included in the placebo video game | Unlike DTx, the intervention’s **therapeutic exercises are not included in the placebo video game** | Active component replaced with inactive/neutral component in digital sham |
| Wijnhoven 2020 | Triple Town | Mindlight | Anxiety of children with an autism spectrum disorder | • Video game based  • Mindlight computer-based intervention that uses visual aids and structured sensory information in training emotion regulation skills (e.g., relaxation).  • Mindlight is aimed at children and is based on principles of CBT and neurofeedback. | • Video game based  • A commercial puzzle game (Triple Town) without any therapeutic elements to help children think strategically to overcome challenges. However, the game does not focus at reducing anxiety levels, which makes ‘Triple Town’ a suitable game for the control condition. | DTx game consists of scary content improving inner strength of participants on the key principles of CBT and neurofeedback**,** whereas **sham control is completely different content** **not intended to impact anxiety** | Active component replaced with inactive/neutral component in digital sham |
| Iacoviello 2018 | Emotional Faces Memory Task training (EFMT) – with neutral shapes | Emotional Faces Memory Task training (EFMT) | Depression | • Computer-based (training)  • Digital intervention designed to enhance cognitive control for emotional information-processing  • Simultaneous activation of dorsolateral prefrontal cortex and amygdala | • Computer-based (training)  • The sham control condition involved a WM training exercise which utilized the same N-back paradigm as EFMT but included neutral shapes as stimuli instead of emotional faces, so no emotion processing or limbic activation was expected.  • Activation of dorsolateral prefrontal cortex only | The sham control condition involved a WM training exercise which utilized the same N-back paradigm as EFMT, but **included neutral shapes as stimuli instead of emotional faces so no emotion processing or limbic activation was expected** | Active component replaced with inactive/neutral component in digital sham |
| Rosa 2017 | Placebo cognitive training | ACTIVATE | ADHD | • A software based computerized cognitive remediation training (CCRT)  • This includes six different games that target neurocognitive functions, such as WM, speed processing, sustained and divided attention, category formation and control inhibition. Participants perform a wide range of cognitive tasks like memorizing sequences, completing patterns, task-switching and assigning objects into categories. | • A computerized placebo training, consisted of the same exposure time of the active training but without any component of neurocognitive functions  • A package composed of educational videos and questions related to school content was developed by a learning tutor and psychologists; the training package considered the academic level of the participants and was hosted on an online platform (Moodle) at the hospital. | ACTIVATE includes CCRT which comprises six different games that target neurocognitive functions whereas digital sham consists of **educational videos and questions related to school content** was developed by a learning tutor and psychologists and hosted on an online platform | Active component replaced with inactive/neutral component in digital sham |
| Scholten 2016 | Rayman 2: The Great Escape | Dojo | Anxiety | • Video game (played on a computer or laptop)  • Dojo incorporates two evidence-based strategies for reducing anxiety symptoms: emotion regulation training and heart rate variability (HRV) biofeedback.  • Played from a first-person perspective.  • During these challenges the player’s heart rate is monitored by a biofeedback system that measures heart rate via sensors attached to the player’s fingers. Heart rate measures are directly read into Dojo so that players’ arousal levels are continuously displayed in the right corner of the video game. To encourage player’s regulation of emotions, each of the game’s challenges become increasingly difficult if the player’s heart rate increases. | • Video game (played on a computer or laptop)  • Rayman is a commercially available platform video game developed by Ubisoft Entertainment S.A.  • Played from a third-person perspective, and the player has control over the camera.  • Rayman takes place in a world called the Glade of Dreams. Rayman must navigate through the world, defeat numerous threatening enemies, and solve puzzles. | • Even though Rayman was not specifically designed for anxiety reduction, it may incorporate some of the more general action mechanisms that have benefits for adolescent’s emotional development more  broadly, and anxiety more specifically  • Unlike Dojo, **control condition does not consist of HRV biofeedback-based cognitive emotion regulation** training to remain calm in stressful situations  • Dojo is played from a first-person perspective, unlike Rayman 2: The Great Escape which is played from a third-person perspective, with control over the camera.  • Dojo takes place in a secret temple hidden underground whereas Rayman takes place in a world called the Glade of Dreams  • In Dojo the player takes the role of a young person who is going to discover a secret temple and encounters different dojo masters, but has to remain calm in stressful situations. In the control game Rayman defeats numerous threatening enemies, and solves puzzles | Active component replaced with another active component in digital sham |
| Schoneveld 2016 | Max and the Magic Marker (Max) | MindLight | Anxiety | • Video game based (computer or laptop)  • Mindlight is based on principles of CBT and neurofeedback.  • Consists of scary content which allows the user to use his own inner strength to overcome his greatest fears | • Video game based (computer or laptop)  • The game has three different worlds inspired by children’s drawings, each with five levels with death traps, monsters, and puzzles. | • Unlike MindLight, the **digital sham does not consist of neurofeedback-based CBT** including various relaxation techniques (e.g., deep breathing, self-talk) while they approach the “fear events” and ‘inner strength’ measuring neurofeedback headset  • The MindLight avatar (little Arty) is in a dark haunted house whereas the Max avatar (“underdog”) has to conquer fearful obstacles like death traps, monsters, and puzzles. | Active component replaced with inactive/neutral component in digital sham |
| de Vries 2015 | Non-adaptive control training (mock-training) | ‘Braingame Brian’, an adaptive WM training and cognitive flexibility | ASD | A computerized executive function (EF)-training with game-elements | • Computer games  • A mock-training consists of all tasks remained at a low, nonadaptive level | Digital sham delivers a mock-training which consists of all tasks remained at a **low, nonadaptive level** | Same game but at a low-level in digital sham group |
| Dovis 2015 | Braingame Brian on placebo mode | Braingame Brian | ADHD | A computerized executive function (EF)-training with game-elements | A computerized WM, inhibition, and cognitive-flexibility all in placebo-mode. | No difference, same components in sham game but all WM, inhibition and cognitive-flexibility tasks were in **placebo mode** | Same game but at a low-level in digital sham group |
| Bikic 2017 | Placebo control arcade game (Tetris) | Scientific Brain Training (SBT) | ADHD | • Video game training  • Computer  • SBT is a computer program that consists of six exercises: Entangled, Figures, Shapes and Colors, Under Pressure, Displaced Characters, Heraldry, and Objects Where are You? | • Tetris game  • Computer  • A video puzzle game that involves manipulating falling shapes | Unlike the DTx, the Tetris game **scenario did not change over the course** of the following sessions. The participants had **to repeat the same task repeatedly.** | Active component in DTx replaced with another active component in digital sham |
| Chacko 2014 | Cogmed Working Memory Training placebo (CWMT placebo) | Cogmed Working Memory Training (CWMT) | ADHD | • Computer  • Computerized WM training program  • Training is supervised by a training aide (typically a parent or guardian) and a certified CWMT coach, who is able to track closely (via online access) each individual’s performance and provide support to the family through weekly coaching interactions (by phone).  • CWMT Active trials are titrated to the capacity of the individual using an adaptive staircase design that adjusts the difficulty of the program on a trial-by-trial basis. | • Computer  • As with CWMT Active, parents in the CWMT Placebo served as training aides, and each family was supported by a coach who utilized comparable support procedure. | Unlike the active condition, **difficulty level was not scaffolded** according to each user’s performance parameters in the placebo condition. | Same game but at a low-level in digital sham group |
| Stasiak 2014 | Placebo program with psychoeducational content (CPE) | The Journey | Depression | • CD ROM (computerized multimedia Flash-based programs)  • CBT-based seven modules guiding self-help intervention, delivered on a CDROM  • An avatar-based fantasy game where avatar follows a narrative of a quest through magical lands and face weekly challenges based on modules targeting CBT restructuring | • CD ROM (computerized multimedia Flash-based programs)  • A computer-administered attention placebo program with psychoeducational content (CPE)  • Consists of 7 modules | •DTx is an avatar-based game targeting CBT restructuring, whereas **sham control simply delivers psychoeducational content (CPE)** | Active component replaced with inactive/neutral component in digital sham |
| Hancock 2015 | Sham training (computerized cognitive training) | Brain HQ (InSight or BrainTwister visual n-back programs) | Multiple Sclerosis (memory training) | • Computer  • Game based  • Processing speed tasks: Two processing speed tasks were employed: PositScience’s Sweep Seeker and Road Tour.  • Two WM tasks were employed: Posit Science’s Master Gardener and the Brain Twister N-Back Task. (Participants in the study played a single modality visual n-back game. The Active Training group’s tasks increased in difficulty) | • Computer  • Game based  • Processing speed tasks: For the sham training group, the games stayed at a simple, introductory level of difficulty.  • WM tasks: The sham training group played a 0-back condition of the game created specifically for this study but modelled after the active group task. | Same components except **lower game difficulty level** in sham control | Same game but at a low-level in digital sham group |
| Van der Molen 2010 | Control training (with no demand on memory capacity) | Odd Yellow WM training (Training A & B) | Intellectual disability | • Computer  • Internet  • Odd Yellow WM training (adaptive and non-adaptive WM training)  • The adaptive training starts with momentary capacity and continuously challenges the WM capacity progressively each session | • Computer  • Internet  • The control training does not place any demand on memory capacity | The control training was nearly identical to Training A but **without placing any demand on memory capacity**. | Active component replaced with inactive/neutral component in digital sham |
| NCT00850447 | Placebo control arcade game (Tetris) | CogPack | Post-traumatic stress disorder | • Computer-based form of cognitive remediation therapy (version 6.0 Marker Software, Mannheim Germany) | • Tetris game  • Computer  • A video puzzle game that involves manipulating falling shapes | • Unlike Tetris, CogPack is a computerized cognitive remediation therapy which does not involve video game  • **Tetris involves using visuospatial and motor abilities, rather than memory and attentional abilities**, and thus lacked the key therapeutic active components for PTSD. | Active component in DTx replaced with another active component in digital sham |
| NCT04574921 | Light therapy system (LTS): Sham setting | 40 Hz Light therapy system (LTS) | Alzheimer’s disease | • Invisible spectral flickering light | • Continuous non-flickering white light | DTx consisted of LTS device set to 40 Hz invisible spectral flicker whereas sham comparator provided LTS device set to continues color matched white light | Active component replaced with inactive/neutral component in digital sham |

ABM: Attention bias modification; ABMT: Attention bias modification training; ADHD: Attention deficit hyperactivity disorder; BAT: Behavioral Avoidance Test; CBT: Cognitive behavioural therapy; CCRT: Computerized cognitive remediation training; CWMT: Cogmed Working Memory Training; DEAL: Depression Alcohol Project; dCBT-I: Digital cognitive-behavioral therapy for insomnia; DLPFC: Dorsolateral prefrontal cortex; DTx: Digital therapeutics; eCBTI: e-aid Cognitive Behavioural Therapy for Insomnia; EEG: Electroencephalogram; EF: Executive function; EFMT: Emotional Faces Memory Task training; HD-ABM: Home-delivered attentional bias modification; HMD: Head mounted display; HRV: Heat rate variability; LTS: Light therapy system; PTSD: Post-traumatic stress disorder; SBT: Scientific brain training; TEC: Therapeutic evaluative training; VR: Virtual reality; VRAT: Virtual Reality Attention Training; WM: Working memory; XR: Extended reality.

Supplementary Table S7: Duration of digital sham

| Study names | Name of digital sham | Name of DTx | Indication | Duration of DTx | Duration of Digital sham treatment | Does duration of Digital sham treatment match with DTx? (Yes/ No) |
| --- | --- | --- | --- | --- | --- | --- |
| Mobile devices- based | | | | | | |
| Bove 2021 (DigCog) | AKL-T09 | AKL-T03 | Multiple sclerosis | 6 weeks  (25 minutes a day, 5 days a week) | 6 weeks  (25 minutes a day, 5 days a week) | Yes |
| Kollins 2020 (STARS-ADHD) | AKL-T09 | AKL-T01 (EndeavorRx) | ADHD | 4 weeks  (25 minutes a day, 5 days a week) | 4 weeks  (25 minutes a day, 5 days a week) ^ø^ | Yes |
| Keefe 2019 (STARS-MDD) | AKL-T09 | AKL-T03 | Major depressive disorder | 6 weeks  (25 minutes a day, 5 days a week) | 6 weeks  (25 minutes a day, 5 days a week) | Yes |
| Teng 2019 | Placebo training | Home-delivered attentional bias modification (HD-ABM) | Generalized Anxiety disorder | 4 weeks  (3 times a day) | 4 weeks  (3 times a day) | Yes |
| Yerys 2019 | AKL-T09 | Project EVO | Autism Spectrum Disorder and ADHD | 4 weeks  (25 minutes each session) ^¤^ | 4 weeks  (25 minutes each session) | Yes |
| Bucci 2018 | ClinTouch app | Actissist | Psychosis | 12 weeks  (6 days a week between 10.00 and 22.00 alongside usual treatment) | 12 weeks  (6 days a week between 10.00 and 22.00 alongside usual treatment) | Yes |
| Deady 2018 | HeadGear lite app | HeadGear | Depression | 4 weeks | 4 weeks | Yes |
| Davies 2017 | Imagination of information about alcohol misuse | Drinks Meter | Risky Drinking | 4 weeks | 4 weeks | Yes |
| Dennis-Tiwary 2017 | Placebo – Attention training application | Personal Zen: Attention bias modification training (ABMT) | Stress and anxiety during pregnancy | 4 weeks | 4 weeks | Yes |
| Giosan 2017 | Placebo app against Dcombat | Dcombat | Depression | 6 weeks  (One module every 10 days) | 6 weeks  (One module every 10 days) | Yes |
| Franklin 2016 | Therapeutic Evaluative Conditioning (TEC) with neutral (or blank) images | Therapeutic Evaluative Conditioning (TEC) | Self-injurious thoughts and behaviors (SITBs) | 4 weeks  (1-2 min to complete a single instance) | 4 weeks  (1-2 min to complete a single instance) | Yes |
| Enock 2014 | Control training (no contingency training) | Attention Bias Modification Training | Anxiety (social anxiety) | 4 weeks  (3 sessions daily) | 4 weeks  (3 sessions daily) | Yes |
| NCT04779372 | Online sleep education/ Sleep Hygiene Education | WeChat app – eCBTI | Insomnia | 6 weeks | 6 weeks | Yes |
| NCT03751280 | PEAR-004 placebo app | PEAR-004 | Schizophrenia | 12 weeks | 12 weeks | Yes |
| NCT02828644 | AKL-T09 | AKL-T01 (EndeavorRx) | ADHD | 4 weeks  (25 minutes a day, 5 days a week) | 4 weeks  (25 minutes a day, 5 days a week) ^µ^ | Yes |
| Web-based | | | | | | |
| Cheng 2021 (SPREAD trial) | Online sleep education/ Sleep Hygiene Education | Sleepio | Insomnia | 6 weeks  (6 sessions typically lasting 20 minutes each) | 6 weeks | Yes |
| Kalmbach 2020 | Online sleep education/ Sleep Hygiene Education | Sleepio | Insomnia in pregnancy | 6 weeks  (6 sessions typically lasting 20 minutes each) | 6 weeks | Yes |
| Espie 2019 (DIALS study) | Online sleep education/ Sleep Hygiene Education | Sleepio | Insomnia | 6 weeks  (6 sessions typically lasting 20 minutes each) | 6 weeks | Yes |
| Batterham 2018 | HealthWatch | FitMindKit | Mood, anxiety, substance use and suicidality | 2 weeks | NR^Δ^ | NR |
| Parks 2018 | Psychoeducation | Happify | Anxiety and depression | 8 weeks | 8 weeks | Yes |
| Denis 2017 | Puzzles | Sleepio | Insomnia | 6 weeks  (20-25 minutes session) | 6 weeks  (20-25 minutes session) | Yes |
| Perry 2017 (TriPoD) | lifeSTYLE | SPARX-R | Depression | 5 weeks (seven modules approximately 25 minutes each) | 5 weeks (seven modules approximately 25 minutes each) | Yes |
| Zwerenz 2017 | Online information about depression | Deprexis | Depression | 12 weeks | 12 weeks | Yes |
| Christensen 2016 (GoodNight Study) | HealthWatch | SHUTiP | Insomnia and depression | 9 weeks  (A 6-week program conducted over a window of 9 weeks) | 9 weeks | Yes |
| Deady 2016 | HealthWatch | DEAL Project (Depression Alcohol) intervention | Depression and Problematic Alcohol Use | 4 weeks | 4 weeks | Yes |
| Pinheiro 2016 | Online sleep education/ Sleep Hygiene Education | Sleepio | Insomnia in patients with lower back pain | 6 weeks  (6 sessions typically lasting 20 minutes each) | 6 weeks | Yes |
| Espie 2012 | Imagery relief therapy (IRT) | Sleepio | Insomnia | 6 weeks | 6 weeks | Yes |
| NCT03724305 (RESIST) | Online sleep education/ Sleep Hygiene Education | Sleepio | Insomnia | 6 weeks  (6 sessions typically lasting 20 minutes each) | 6 weeks | Yes |
| NCT03613519 | Online sleep education/ Sleep Hygiene Education | SHUTi | Insomnia | 6-8 weeks | 6-8 weeks | Yes |
| NCT03322774 (STRIDE) | Online sleep education/ Sleep Hygiene Education | Sleepio | Insomnia | 6 weeks  (6 sessions typically lasting 20 minutes each) | 6 weeks | Yes |
| NCT04112225 | Psychoeducation | Happify | Loneliness | 8 weeks | 8 weeks | Yes |
| Extended reality | | | | | | |
| Bentz 2021 | Google Street View app | Easy Heights | Fear of heights | 2 weeks  [A single 1-h app use (phase 1) and after additional repeated (6 × 30 min) app use at home (phase 2)] | 2 weeks  [A single 1-h app use (phase 1) and after additional repeated (6 × 30 min) app use at home (phase 2)] | Yes |
| Garcia 2021 | EaseVRx headset without active intervention | EaseVRx headset with active intervention | Chronic Lower back Pain | 8 weeks  (56 sessions, with a duration between 2.5 and 5 minutes) ^¶^ | 8 weeks  (56 sessions, with a duration between 2.5 and 5 minutes) | Yes |
| Smeijers 2019 | XR placebo game | Virtual Reality Game for Aggression Impulsive Management (VR-GAIME) | Aggression regulation problems | 12 weeks | 12 weeks | Yes |
| McEwen 2014 | Control, virtual reality system (IREX) | Virtual Reality system (IREX) | Stroke | 3-weeks  (10 to 12 thirty-minute daily sessions) | 3-weeks  (10 to 12 thirty-minute daily sessions) | Yes |
| In 2012 | Control (sham program) | Virtual reality reflection therapy program | Chronic stroke | 4-weeks  (30 minutes as day, 5 days a week) | 4-weeks  (30 minutes as day, 5 days a week) | Yes |
| NCT04714645 | Sham XR training | Oculus Rift (HMD VR + Training) | Stroke | 0.7 week (5 days, 1 session a day for 30 minutes) | 0.7 week (5 days, 1 session a day for 30 minutes) | Yes |
| NCT04443127 | Motor-Relearning Program | Virtual Reality Training Session- Game-Based Rehabilitation | Stroke | 4 weeks  (16 sessions, comprising of 45 minutes, 4 times/week) | 4 weeks  (16 sessions, comprising of 45 minutes, 4 times/week) | Yes |
| NCT04225884 | Sham XR software for chronic pain | Virtual Reality Software for Chronic Pain (VIRPI) | Chronic Lower back Pain | 6-8 weeks | 6-8 weeks | Yes |
| NCT04139564 | EaseVRx headset without active intervention | EaseVRx headset with active intervention | Chronic Lower back Pain | 8 weeks  (7 weekly modules each approximately 5 minutes in duration, for a total of 56 modules across the program) | NR | NR |
| NCT03135418 | XR program only focusing on visual scenes without upper extremity interaction | Oculus Rift and Leap motion | Stroke | 6 weeks  (Sessions on each Monday, Wednesday, and Friday at same time for each patient). | 6 weeks  (no further details available) | Yes |
| NCT02066116 | Sham virtual rehabilitation education | Kinect-based Rehabilitation | Stroke | 1.4 weeksˠ (30 min/session) | 1.4 weeksˠ | Yes |
| Wearable | | | | | | |
| Cimenser 2021 | GENUS (gamma entrainment using sensory stimuli): Sham Settings | GENUS (gamma entrainment using sensory stimuli) or synchronized 40 Hz gamma oscillation | Alzheimer’s disease | 1-h daily sessions over a 6-month period | 1-h daily sessions over a 6-month period | Yes |
| Pasula 2020 | NerivioMigra app (REN device) - Sham stimulation | NerivioMigra app (REN device) | Acute migraine | 45 min | 45 min | Yes |
| Schenker 2019 | Relivion Device- Sham stimulation | Relivion | Migraine | 1 hour | 1 hour | Yes |
| NCT03631550 (The RIME Study) | Relivion Device- Sham stimulation | Relivion | Migraine | 1 hour | 1 hour | Yes |
| NCT03185559 | Relivion Device- Sham stimulation | Relivion | Migraine | 1 hour | 1 hour | Yes |
| Computer-based | | | | | | |
| Sosnowski 2021 | Control video game | Lookware™ | Autism Spectrum Disorder | 6 weeks | 6 weeks ^ô^ | Yes |
| Wijnhoven 2020 | Triple Town | Mindlight | Anxiety of children with an autism spectrum disorder | 6 weeks  (1 h per week) | 6 weeks  (1 h per week) | Yes |
| Iacoviello 2018 | Emotional Faces Memory Task training (EFMT) – with neutral shapes | Emotional Faces Memory Task training (EFMT) | Depression | 6 weeks  (Three times per week) | 6 weeks  (Three times per week) | Yes |
| Rosa 2017 | Placebo cognitive training | ACTIVATE | ADHD | 12 weeks  (4 sessions/week; duration 30 min) | 12 weeks  (4 sessions/week; duration 30 min) | Yes |
| Scholten 2016 | Rayman 2: The Great Escape | Dojo | Anxiety | 3 weeks  (6 sessions of one hour spread over three weeks) | 3 weeks  (6 sessions of one hour spread over three weeks) | Yes |
| Schoneveld 2016 | Max and the Magic Marker (Max) | MindLight | Anxiety | ~ 2.5 weeks  (5 one-hour sessions, scheduled twice a week) | ~ 2.5 weeks  (5 one-hour sessions, scheduled twice a week) | Yes |
| de Vries 2015 | Non-adaptive control training (mock-training) | ‘Braingame Brian’, an adaptive working memory (WM) training and cognitive flexibility | Autism Spectrum Disorder | 6-weeks | 6-weeks | Yes |
| Dovis 2015 | Braingame Brian on placebo mode | Braingame Brian | ADHD | 5 weeks  (25 sessions, each session for about 35–50 minutes) | 5 weeks  (25 sessions, each session for about 35–50 minutes) | Yes |
| Bikic 2017 | Placebo control arcade game (Tetris) | Scientific Brain Training (SBT) | ADHD | 7 weeks  (Half an hour a day, 5 days a week) | 7 weeks  (Half an hour a day, 5 days a week) | Yes |
| Chacko 2014 | Cogmed Working Memory Training placebo (CWMT placebo) | Cogmed Working Memory Training (CWMT) | ADHD | 5 weeks  (Cognitive remediation training: 5-week, 5days/week  Parent training: 2.0 hour, weekly, group sessions that focus on behavioural management procedures) | 5 weeks  (Cognitive remediation training: 5-week, 5days/week  Parent training: 2.0 hour, weekly, group sessions that focus on behavioural management procedures) | Yes |
| Stasiak 2014 | Placebo program with psychoeducational content (CPE) | The Journey | Depression | 4-8 weeks  (Program consists of 7 modules requiring sequential completion) | 4-8 weeks  (Program consists of 7 modules requiring sequential completion) | Yes |
| Hancock 2015 | Sham training (computerized cognitive training) | Brain HQ (InSight or BrainTwister visual n-back programs) | Multiple Sclerosis (memory training) | 6 weeks  (6 days per week, for 30-minute intervals) | 6 weeks  (6 days per week, for 30-minute intervals) | Yes |
| Van der Molen 2010 | Control training (with no demand on memory capacity) | Odd Yellow Working Memory (WM) training (Training A & B) | Intellectual disability | 5 weeks  (3 times a week for 6 mins, 15 sessions across 5 weeks) | 5 weeks  (3 times a week for 6 mins, 15 sessions across 5 weeks) | Yes |
| NCT00850447 | Placebo control arcade game (Tetris) | CogPack | Post-traumatic stress disorder | 10 weeks  (60-minute sessions) | 10 weeks  (30-minute sessions) | Overall duration (no. of weeks) is the same, however per session gameplay time is not |
| NCT04574921 | Light therapy system (LTS): Sham setting | 40 Hz Light therapy system (LTS) | Alzheimer’s disease | 1 week | 1 week | Yes |

ADHD: Attention deficit hyperactivity disorder; CWMT: Cognitive working memory training; DEAL: Depression-Alcohol (DEAL) Project; DTx: Digital therapeutic; eCBTI: e-aid Cognitive Behavioural Therapy for Insomnia; EFMT: Emotional Faces Memory Task training; HD-ABM: Home-delivered attentional bias modification; min: Minute; NR: Not reported; SBT: Scientific brain training; STARS-ADHD: Software Treatment for Actively Reducing Severity of ADHD; STARS-MDD: Software Treatments for Actively Reducing Severity of Cognitive Deficits in major depressive disorder; TEC: Therapeutic Evaluative Conditioning; WM: Working memory; XR: Extended reality.

^Ø^: During the intervention period (days 1–28), patients were instructed to use AKL-T01 or the control at home for 5 sessions per day (total time on task about 25 min), 5 days per week, for 4 weeks or the control for 25 min per day, 5 days per week, for 4 weeks [pediatric ADHD] 25 minutes/day, 5 days/week, for 6 weeks

¤: Five 5-min runs for one multi-tasking session or one 25-min session for one education session. Children are asked to complete 20 sessions (five times a week for 4 weeks), though the treatment could be accessed up to seven times a week for a total of 28 sessions

µ: During the intervention period (days 1–28), patients were instructed to use AKL-T01 or the control at home for 5 sessions per day (total time on task about 25 min), 5 days per week, for 4 weeks or the control for 25 min per day, 5 days per week, for 4 weeks [pediatric ADHD] 25 minutes/day, 5 days/week, for 6 weeks

^Δ^: This is used as an attention control. Therefore, duration must be similar to the DTx used. However, in general HealthWatch versions commonly available have duration 9-12 weeks. The study has mentioned evidence of placebo effect of HealthWatch from literature (i.e., Glozier et al., 2013). Glozier et al., 2013 used 12 weeks version of HealthWatch.

^¶^: The standardized 56-day program delivers a multifaceted combination of skills training through a prescribed sequence of daily virtual experiences. Each XR experience lasts between 2 and 16 minutes, with an average duration of 6 minutes of treatment time.

^Ô^: The number of sessions per week and total hours of gameplay were similar across conditions (p > 0.05).

ˠ: Mentioned as 10 days in study, converted to weeks i.e., 1.4 weeks
